# Lactic Acid Bacteria as a Live Delivery System for the *in situ* Production of Nanobodies in the Human Gastrointestinal Tract

**DOI:** 10.3389/fmicb.2018.03179

**Published:** 2019-01-08

**Authors:** Beatriz del Rio, Begoña Redruello, Maria Fernandez, M. Cruz Martin, Victor Ladero, Miguel A. Alvarez

**Affiliations:** Department of Technology and Biotechnology of Dairy Products, Dairy Research Institute (IPLA-CSIC), Madrid, Spain

**Keywords:** VHH, nanobodies, lactic acid bacteria, *in situ* delivery, gastrointestinal tract, prophylaxis, therapy

## Abstract

Lactic acid bacteria (LAB) are among the most widely used microorganisms in food fermentation. However, some LAB species can also be used as live vehicles for the *in situ* delivery of therapeutic molecules to the mucosa of the human gastrointestinal tract (GIT). Many LAB species have ‘qualified presumption of safety’ status and survive passage through the GIT. Indeed, some are part of the usual GIT microbiota. These are appropriate candidates for the *in situ* production of recombinant prophylactic and therapeutic proteins. Live recombinant LAB that produce microbial antigens have been shown to elicit an immune response that confers protection against the corresponding pathogens; these LAB could therefore be used as oral vaccines. In addition, some LAB have been genetically engineered to produce therapeutic, neutralizing antibodies. The variable domain of heavy-chain-only antibodies from camelids – known as VHH antibodies or nanobodies – has peculiar properties (nanoscale size, robust structure, acid resistance, high affinity and specificity, easily produced in bacteria, etc.) that make them ideal choices as LAB-produced immunotherapeutic agents. The present review examines the advantages offered by LAB for the *in situ* production of therapeutic proteins in the human GIT, discusses the use of *in situ* produced VHH antibody fragments, and assesses the usefulness of this strategy in the treatment of infectious and non-infectious gastrointestinal diseases.

## Introduction

New therapeutic strategies are needed if we are to better face the challenges posed by cancer, resistance to antibiotics, and viral infections. The development of systems that allow drugs to be more precisely delivered to their target organs, and that better control their release, is a major goal (Wells, 2011; Hosseinidoust et al., 2016); non-specific drug delivery can be associated with toxic side effects in non-target tissues and organs.

It has been proposed that live bacteria be used as vectors for the *in situ* delivery of recombinant proteins for prophylactic and therapeutic purposes (Medina and Guzman, 2001; Wells and Mercenier, 2008; Cano-Garrido et al., 2015; Hosseinidoust et al., 2016; Ding et al., 2018). This strategy should be inexpensive since bacteria are easy to grow, the pharmaceutical production and purification of the active agent are avoided, and degradation problems (which are particularly severe in the gastrointestinal tract [GIT]) can be overcome (Wells, 2011; Wang et al., 2016). The producing bacteria can also be lyophilized, avoiding the need to maintain a cold chain (Pant et al., 2006). Attenuated pathogenic bacteria were originally proposed for use in such systems, but lactic acid bacteria (LAB) quickly became recognized as ideal candidates, especially for the prevention and treatment of mucosal diseases (Cano-Garrido et al., 2015; Wang et al., 2016).

## Advantages of Lab as Live Vectors for the *In Situ* Production of Therapeutic Proteins

The LAB form a heterogeneous group of Gram-positive bacteria that include technologically important species of the genera *Lactobacillus*, *Lactococcus*, *Streptococcus, Leuconostoc*, *Oenococcus*, and *Pediococcus*. They are mainly characterized by their ability to produce lactic acid from sugar – an ability long exploited by humans to produce fermented foods such as cheese, yoghurt, sausage, wine, sauerkraut, and pickled vegetables. Such foods can be safely consumed long after they are made and may even be of enhanced nutritional value. The presence of LAB in foods and their lack of pathogenic traits or association with any foodborne disease have led to their being regarded as safe. In fact, most LAB species are ‘generally regarded as safe’ (GRAS) by the US Food and Drug Administration (FDA) and have been awarded ‘qualified presumption of safety’ (QPS) status by the European Food Safety Authority (EFSA).

LAB occupy a wide range of niches. Some species are found in milk and in plants, while others are common members of the endogenous microbiota associated with the oral, urogenital, and GIT mucosae in animals and humans (Klaenhammer et al., 2002). Certain LAB strains play a role in controlling the intestinal microbiota, restoring the intestinal barrier function, and alleviating the inflammatory responses associated with GIT disease. This has fomented their use in the treatment and prevention of various intestinal disorders (Ouwehand et al., 2002; Isolauri et al., 2004; Gareau et al., 2010). Certain LAB groups, such as the lactobacilli, are important members of the endogenous microbiota of human mucosal surfaces, where they play crucial roles in homeostasis, providing the protection against pathogenic bacteria and stimulating the immune system (Isolauri et al., 2004; Bernardeau et al., 2008). Given their beneficial nature, LAB species such as *Lactobacillus acidophilus*, *Lactobacillus casei*, and *Lactobacillus rhamnosus*, which meet the definition of probiotics (FAO/WHO, 2006), have been added to food to provide consumers with their health benefits (Saxelin et al., 2005; Gareau et al., 2010; Salminen et al., 2010). Indeed, LAB, and lactobacilli in particular, have become a focus of interest for use in human and animal biomedical applications (Wells and Mercenier, 2008; Behnsen et al., 2013; Cano-Garrido et al., 2015; Wang et al., 2016).

LAB are reported to be ideal candidates as live delivery vehicles for releasing therapeutic and prophylactic molecules directly at the oral, nasal, and genital mucosae and to be a realistic option for the treatment of human and animal diseases (Cano-Garrido et al., 2015; Piñero-Lambea et al., 2015; Wang et al., 2016). The safety status of the LAB, the ability of some strains to survive passage through the GIT (Vesa et al., 2000), and the capacity of some species (e.g., *L. rhamnosus*, *Bifidobacterium animalis*, and *Lactobacillus plantarum*) to remain viable in the GIT for a period of time render food-grade LAB ideal vehicles for delivering and even producing therapeutic molecules *in situ* at the GIT mucosa (Daniel et al., 2011; Wang et al., 2016). The absence of lipopolysaccharides (LPSs) in their cell walls (which is not the case in Gram-negative bacteria such as *Escherichia coli*) is a further advantage, allowing them to be administered orally without the risk of endotoxic shock (Szatraj et al., 2017). In addition, the oral administration of therapeutic molecules *via* live recombinant LAB is a suitable alternative to invasive administration methods, for example, parenteral or subcutaneous injection, avoiding their potential side effects. Further, it circumvents the degradation of orally administered naked molecules in the digestive tract and ensures the production of the therapeutic protein at the GIT mucosa (Wang et al., 2016). Moreover, the *in vivo* synthesis of the therapeutic molecule reduces the dose required when compared to systemic or subcutaneous treatment (Steidler et al., 2000; Cano-Garrido et al., 2015).

In recent decades, much effort has gone into the genetic manipulation of LAB with the aim of producing recombinant therapeutic molecules (García-Fruitós, 2012; Cano-Garrido et al., 2015). Tools that allow cloning, the modulation of expression, and even the localization of recombinant proteins are now available (de Ruyter et al., 1996; Martin et al., 2000, 2011; Hanniffy et al., 2004; Benbouziane et al., 2013; Linares et al., 2014; Linares et al., 2015; Michon et al., 2016). Recombinant proteins can be engineered to be secreted into the extracellular environment or to be secreted and then anchored on the bacterial surface. Proteins to be secreted must have an N-terminus signal peptide recognized by the bacterial secretion machinery. One of the secretion mechanisms most studied in genetic engineering is the Sec-dependent pathway (Mathiesen et al., 2008). This drives the translocation of the precursor protein (i.e., the signal peptide plus the mature protein) across the plasma membrane. Either during or after translocation, a signal peptidase cleaves off the signal peptide and the mature protein is released into the extracellular environment (Schneewind and Missiakas, 2014). Different signal peptides have been exploited for engineered secretion in LAB, such as that associated with the major lactococcal secreted protein Usp45 (Dieye et al., 2001), the *Lactobacillus brevis* S-layer protein (SlpA) (Oh et al., 2007), the *Streptococcus pyogenes* M6 protein (Hols et al., 1997), and the *Lactobacillus crispatus* aggregation-promoting factor (APF) (Martin et al., 2011; Pant et al., 2011; Gunaydin et al., 2014), among others (Mathiesen et al., 2008). Secreted recombinant proteins can also be engineered by the translational fusion of an anchor peptide (displayed on the bacterial surface) *via* covalent or non-covalent bonding (Desvaux et al., 2006; Zadravec et al., 2015; Mao et al., 2016; Michon et al., 2016). Indeed, several anchoring peptides derived from surface-exposed proteins of Gram-positive bacteria have been used to bind secreted recombinant proteins to the surface of LAB – either to the plasma membrane or to the cell wall components (Michon et al., 2016). The signals used to anchor the protein to the cell membrane include N-terminal transmembrane helix anchors, for example, the transmembrane domain of the PgsA protein from *Bacillus subtilis* (Narita et al., 2006), and lipoprotein anchor domains, for example, the anchor domain of lactococcal basic membrane protein A (BmpA) (Zadravec et al., 2014). Recombinant proteins can be covalently anchored to the bacterial cell wall, which is usually achieved by fusing the target protein to an LPXTG-type cell wall-sorting signal. This consists of an LPXTG motif followed by a C-terminal hydrophobic domain and a positively charged tail that adequately orientates the protein in the membrane, allowing a membrane-anchored sortase to cut a peptide bond of the LPXTG motif and catalyze covalent bonding with the peptidoglycan (Schneewind et al., 1993; Siegel et al., 2017). The LPXTG anchors used include the cell-wall anchor of the *S. pyogenes* M6 protein (Dieye et al., 2001; Cortes-Perez et al., 2003), the anchor sequence of proteinase P of *Lactobacillus zeae* (Kruger et al., 2002; Martin et al., 2011), and the LPXTG anchor of the cell wall surface adherent protein Lp_2578 produced by *L. plantarum* WCFS1 (Fredriksen et al., 2010). Recombinant proteins may also be non-covalently attached to the cell wall of LAB by fusing them to different cell wall-binding domains such as the anchoring system of the APF of *L. crispatus* (Martin et al., 2011), the lysin motif (LysM) (Fredriksen et al., 2012), and the C-terminal region of S-layer proteins (Hu et al., 2011). Thanks to these genetic tools, a large number of recombinant LAB strains have been designed that produce therapeutic or prophylactic proteins (Wells and Mercenier, 2008; Bermudez-Humaran et al., 2011; Daniel et al., 2011; Cano-Garrido et al., 2015; Wang et al., 2016).

## Recombinant Lab that Produce Therapeutic Molecules

Some LAB are natural components of the GIT microbiota. Strains such as *L. rhamnosus* GG and *B. animalis* BB12 have been used as probiotics for modulating the GIT microbiota and even to treat some intestinal disorders (Gerritsen et al., 2011). The earliest use of therapeutic molecule-producing recombinant LAB was in the treatment of GIT inflammatory diseases and bacterial and viral infections. The literature now contains many articles describing LAB genetically engineered to produce therapeutic proteins for use in the treatment of gastrointestinal disease (Bermudez-Humaran et al., 2011; Cano-Garrido et al., 2015; Mays and Nair, 2018).

### Recombinant LAB for Use Against GIT Inflammatory Diseases

Many recombinant LAB have been developed to fight inflammatory bowel disease (IBD), a group of disorders that cause chronic inflammation in different parts of the GIT. Crohn’s disease and ulcerative colitis are the two most important types. Almost two decades ago, a recombinant strain of *Lactococcus lactis* was engineered to secrete the anti-inflammatory interleukin 10 (IL-10) (Steidler et al., 2000), and when administered orally, it reduced colitis in two mouse models (mice treated with dextran sulfate sodium [DDS] and IL-10^−/−^ mice). The success obtained led the authors to continue their research with the aim of performing clinical trials, and they developed a biocontained *L. lactis* producing IL-10 in which the host thymidylate synthase gene (*thyA*) was replaced by a synthetic human *IL-10* gene. Since *thyA* is essential for the growth of *L. lactis*, the viability of the biocontained strain depended on the presence of thymidine or thymine (Steidler et al., 2003). A Phase I clinical trial involving patients with Crohn’s disease reported reduced disease activity and confirmed the strain’s biocontainment, safety, and tolerability (Braat et al., 2006).

Other recombinant LAB strains designed for the oral delivery of anti-inflammatory molecules to the gut have also been reported to prevent or reduce inflammation in different murine models of colitis (Cano-Garrido et al., 2015; Wang et al., 2016). For example, *L. lactis* strains were engineered to produce the immunomodulatory low calcium response V (LcrV) protein of *Yersinia pseudotuberculosis* (Foligne et al., 2007), the cytoprotective trefoil factor (TFF) (Vandenbroucke et al., 2004), the immunosuppressive cytokine interleukin 27 (IL-27) (Hanson et al., 2014), insulin-like growth factor I (IGF-I) (Liu et al., 2016), heat shock protein 65 (Hsp65) (Gomes-Santos et al., 2017), and serine protease inhibitors (Bermudez-Humaran et al., 2015). Recombinant *L. casei* producing superoxide dismutase has also been designed (Watterlot et al., 2010).

Celiac disease, an autoimmune disorder of genetically pre-disposed individuals, also causes bowel inflammation after the ingestion of gluten. The oral administration of live recombinant LAB producing prolyl endopeptidases – enzymes that degrade the immunotoxic peptides of gluten – might be a way of preventing an inflammatory response (Alvarez-Sieiro et al., 2014). A generated food-grade recombinant *L. casei* strain was reported to stably and constitutively secrete a prolyl endopeptidase able to break down the 33-mer peptide of gluten (one of the most immunotoxic). The strain also resisted deactivation under simulated gastrointestinal conditions in an *in vitro* model with its ability to secrete the enzyme intact (Alvarez-Sieiro et al., 2014).

### Recombinant LAB as Live Mucosal Vaccines

One of the most extended uses of recombinant LAB that produce therapeutic or prophylactic molecules is as live mucosal vaccines (Wells and Mercenier, 2008; Wyszynska et al., 2015; Szatraj et al., 2017). In addition to the already mentioned advantages of LAB as live mucosal delivery vehicles, these microorganisms have additional properties that render them good choices as live vaccine vectors for mucosal vaccination. For instance, some LAB strains show innate immunoadjuvant properties that enhance the immune response provoked by the desired heterologous immunogenic molecule (Hanniffy et al., 2004; Mohamadzadeh and Klaenhammer, 2008; Bermudez-Humaran et al., 2011). In addition, they have the potential to elicit antigen-specific cellular, mucosal, and systemic humoral immune responses (del Rio et al., 2008, 2010; Wells and Mercenier, 2008; Bermudez-Humaran et al., 2011). Further, live recombinant LAB allow for immunization *via* the oral route, enabling the production *in vivo* of the therapeutic or prophylactic molecules at the GIT mucosa and avoiding the degradation of the treatment molecule in the harsh environment of the stomach (Wang et al., 2016). Moreover, administration is non-invasive and can be performed by personnel with relatively low-level training. There are neither any needles nor syringes to dispose of. It is therefore an ideal strategy for large-scale immunization programs (del Rio et al., 2014). Finally, the possibility of storing recombinant lyophilized LAB avoids the need for a cold chain, making these types of vaccine suitable for use in developing countries.

#### LAB for Active Immunization

Recombinant LAB that produce microbial antigens have been proposed as oral vaccines against a range of pathogens. Back in 1993, *L. lactis* was engineered to express fragment C of the tetanus toxin (TTFC) (Wells et al., 1993). Further studies showed that the administration of TTFC-expressing recombinant *L. lactis via* either the nasal (Norton et al., 1997) or the oral route (Robinson et al., 1997) protected mice against a lethal challenge with tetanus toxin. Since then, a large number of recombinant LAB strains have been generated as potential mucosal vaccines to prevent infections caused by other pathogens (Wells, 2011; Wang et al., 2016). Several recombinant LAB orally administered in appropriate animal models were reported to confer *in vivo* protection against infections of the GIT caused by *Helicobacter pylori* (Gu et al., 2009; Hongying et al., 2014; Li et al., 2014), enterohemorrhagic *Escherichia coli* (EHEC) (Ahmed et al., 2014), enterotoxigenic *E. coli* (ETEC) (Liu et al., 2009; Wei et al., 2010; Wen et al., 2012), *Clostridium difficile* (Yang et al., 2013; Guo et al., 2015), *Salmonella enterica* (Kajikawa et al., 2007), rotavirus (Marelli et al., 2011), the yeast *Candida albicans* (Shibasaki et al., 2014), and even parasites such as *Plasmodium yoelii* (Zhang et al., 2005) and *Giardia lamblia* (Lee et al., 2009). Peptide allergen-producing LAB have also been proposed as a therapeutic option for the treatment of peanut (Ren et al., 2014) and cow’s milk allergies (Adel-Patient et al., 2005).

#### LAB for Passive Immunization

LAB might also be used to produce antibodies to generate passive immunity. Passive immunity, which involves the transfer of ready-to-act antibodies, occurs naturally when maternal antibodies are transferred to the fetus through the placenta, and during lactation. However, it can be artificially induced by transferring large numbers of pathogen- or toxin-specific antibodies to a non-immune individual through blood products, for example, in immunoglobulin or antiserum therapy. LAB can be used as a vehicle for passive immunization by engineering them to produce specific antibodies. LAB producing single-chain variable fragment (scFv) antibodies (chimeric molecules that fuse two different regions of both heavy and light immunoglobulin chains) (Harmsen and De Haard, 2007) have been developed in an attempt to provide passive protection against different diseases of the GIT and other organs (Shiroza et al., 2003; Giomarelli et al., 2004; Monedero et al., 2004; Chancey et al., 2006; Marcotte et al., 2006; Yuvaraj et al., 2008; Wells, 2011; Thueng-in et al., 2014; Michon et al., 2015; Marcobal et al., 2016; Wang et al., 2016). After being orally administered in the corresponding animal model, passive immunoprotection developed against gastrointestinal infections caused by *C. albicans* (Oggioni et al., 2001), *Streptococcus mutans* (Kruger et al., 2002, 2005), norovirus (Hoang et al., 2015), and against anthrax edema toxin (Andersen et al., 2011).

## VHH Antibody Fragments

In addition to conventional immunoglobulin G (IgG) antibodies, the serum of llamas, camels, and dromedaries has antibodies with two identical heavy chains but no light chains (Hamers-Casterman et al., 1993; Muyldermans, 2013). The variable domains of these heavy chains are recognized as a new class of antibody fragment: VHH antibody fragments (also known as nanobodies or single-domain antibodies [sdAbs]; hereafter referred to simply as VHH). These have many advantages over conventional monoclonal antibodies (mAb) and scFv, which has encouraged research into their use possible clinical agents, either in purified form or delivered by VHH-producing live systems.

### Advantages of VHH Over Conventional Antibodies

Despite their small size – 12–15 kDa compared to the 150 kDa of traditional antibodies – VHH can recognize and bind to haptens, oligopeptides, and proteins. Indeed, they are the smallest fragments that retain the full antigen-binding capacity of antibodies. Their small size and structural properties allow them to behave like drugs and invest them with unique advantages over mAb for use in biotechnological and biomedical applications (Harmsen and De Haard, 2007; Goldman et al., 2017; Eden et al., 2018). For example, VHH are of low immunogenicity because of their notable sequence similarity with the VHIII subset family of human VH (Cortez-Retamozo et al., 2002; Su et al., 2002; Baral et al., 2006). They also show good solubility and little tendency to aggregate due to the presence of polar and charged amino acid residues (Siontorou, 2013); they exhibit potent-binding capacity (Muyldermans et al., 2001) and have a high affinity for their antigens (Muyldermans et al., 2001). VHH antibody fragments also show remarkably high-conformational stability and can fully reverse the unfolding process induced by denaturing agents and conditions (such as those encountered in the stomach). They can also resist high pressures and urea and guanidinium chloride concentrations (Dumoulin et al., 2002), are extremely stable at high temperatures (below 50 to over 80°C) (van der Linden et al., 1999; Dumoulin et al., 2002), and remain functional under low pH conditions (as low as pH 2) (van der Vaart et al., 2006). Protein engineering has improved the refolding capacity of VHH and therefore their stability under such denaturing conditions (Goldman et al., 2017).

In addition to the conformational stability of VHH, the paratope of these antibody fragments (i.e., the region of the antibody that binds to an epitope) occupies such a small part of the antibody that the recognition of hidden regions of epitopes, hardly accessible to mAb, becomes possible (Muyldermans et al., 2001). Further, and in contrast to mAb, VHH can act as potent and specific enzyme inhibitors since they can access protein cavities and bind to the active site (Lauwereys et al., 1998). Moreover, VHH penetrate tissues much better than mAb and can therefore target solid tumors and metastatic lesions much more effectively (Cortez-Retamozo et al., 2002).

The single-domain nature of VHH allows for the easy cloning of VHH-encoding genes, as well as the generation of multimeric VHH using the same or different building blocks. Multivalent VHH are created when two or more identical target-binding VHH are combined. In addition, multiparatopic or multispecific VHH can be created by combining different VHH targeting different antigens or different epitopes of the same antigen (Els Conrath et al., 2001; Harmsen and De Haard, 2007; Hultberg et al., 2011). Increasing the valency and specificity of VHH enhances their therapeutic efficacy, potency, and cross reactivity over that of monovalent and monospecific VHH (Hultberg et al., 2011; Huet et al., 2014).

These beneficial properties of VHH have boosted their use in the detection of toxins in foods (Xu et al., 2015; Wang et al., 2018), in disease diagnosis (De Meyer et al., 2014; Hu et al., 2017; Leow et al., 2017), in pathogen detection (Saerens et al., 2008; Abbady et al., 2012), and in the treatment of different diseases in humans (Hu et al., 2017) and animals (Harmsen et al., 2009; Liu et al., 2015). The following lines focus on their use in the treatment of non-infectious and infectious diseases of the human GIT.

### VHH as Immunotherapeutic Agents Against Diseases of the GIT

VHH have been used to combat GIT inflammatory diseases, infections, and cancers (Steeland et al., 2016).

#### VHH Against Gastrointestinal Inflammatory Diseases

Over the last decade, interest has increased in developing VHH antibodies that target proteins involved in inflammation as a means of treating gastrointestinal inflammatory diseases, including IBD (Steeland et al., 2016). Bradley et al. (2015) generated a biparatopic VHH against the chemokine receptor CXCR2, a G-protein-coupled receptor that plays an important role in mediating the chemotaxis of neutrophils to sites of inflammation, which is involved in a variety of acute and chronic inflammatory diseases, including IBD, asthma, fibrosis, psoriasis, and rheumatoid arthritis (Stadtmann and Zarbock, 2012). This recognized two different epitopes, allowing it to act as an inverse agonist and inhibitor of CXCR2 signaling *in vitro* – a promising strategy for treating inflammatory diseases.

#### VHH Against Gastrointestinal Bacterial and Viral Infections

VHH have also been directed against bacterial pathogens and their toxins. The intensive clinical and agricultural use of antibiotics has favored the emergence of multi-resistant pathogens that cause difficult-to-treat or even un-treatable healthcare-associated and community-acquired infections (Watkins and Bonomo, 2016; Frieri et al., 2017). There is therefore an urgent need to develop non-antibiotic antimicrobial agents to combat these pathogens. VHH have been designed to target different bacterial pathogens of the gut (or their toxins) (Steeland et al., 2016), for example, against *S. mutans*, the main cause of dental caries (Nakano et al., 2010). The oral administration of VHH targeting streptococcal antigen I/II (SA I/II), a cell wall-anchored adhesin present on the bacterial surface, reduced the development of smooth surface caries in a rat-desalivated caries model, probably by blocking the binding of the bacteria to the enamel pellicle (Kruger et al., 2006). VHH have also been developed against *Campylobacter jejuni*, a zoonotic pathogen that causes foodborne gastroenteritis worldwide (Kaakoush et al., 2015). An orally administered pentameric VHH directed against the flagella of this pathogen (a major virulence factor) reduced its motility *in vitro* and significantly reduced colonization in infected chickens (Riazi et al., 2013). VHH have also been raised against *H. pylori*, a common pathogen that causes gastritis, gastric and duodenal ulcers, gastric cancer, and other gastrointestinal diseases (Venerito et al., 2015). Some VHH target an important virulence factor, in this case the pathogen’s urease enzyme (Ardekani et al., 2013; Hoseinpoor et al., 2014). This hydrolyses urea into ammonia, helping to neutralize the gastric acid in the bacteria’s environment and aiding their colonization of the epithelium (Marshall et al., 1990; Olivera-Severo et al., 2017). These neutralizing VHH were resistant to pepsin and trypsin, an important trait for any VHH to be used in the treatment of *H. pylori* infection.

Other VHH antibodies have also been generated to target the botulinum neurotoxins of *Clostridium botulinum* (Dong et al., 2010; Mukherjee et al., 2012; Bakherad et al., 2013; Yao et al., 2017), the shiga toxins produced by shiga toxin-producing *E. coli* strains (STEC) (Tremblay et al., 2013; Mejias et al., 2016), and toxin A (TcdA), toxin B (TcdB), and binary toxin (CDT) of *C. difficile* (Hussack et al., 2011; Yang et al., 2014; Unger et al., 2015).

VHH have also shown a promise against human viral diseases (Vanlandschoot et al., 2011; Wu et al., 2017). Some have been designed to block the entry of viruses into the host cells by targeting the viral envelope proteins, while others target essential intracellular viral proteins (Wu et al., 2017). These agents have shown a particular promise against rotavirus and norovirus (Ghosh et al., 2018), the most common causes of severe acute gastroenteritis in children (Riera-Montes et al., 2017). Several VHH generated against Group A rotavirus (RVA) strains were shown to neutralize rotavirus *in vitro* and provided different degrees of *in vivo* passive protection against rotavirus challenge when orally administered in a neonatal mouse model (van der Vaart et al., 2006; Garaicoechea et al., 2008; Gómez-Sebastián et al., 2012; Tokuhara et al., 2013; Maffey et al., 2015). They also provided protection against human RVA-induced diarrhea in gnotobiotic piglets (Vega et al., 2013). The therapeutic efficacy of an anti-rotavirus VHH known as ARP1 (ARP: anti-rotavirus protein) has been demonstrated in a randomized placebo-controlled clinical trial (Clinicaltrials.gov number: NCT01259765). The oral administration of purified ARP1 to children with severe rotavirus-associated diarrhea reduced the stool output (Sarker et al., 2013). VHH have also been raised against the two major norovirus genogroups (GI and GII) (Garaicoechea et al., 2015; Koromyslova and Hansman, 2015, 2017), with some showing promise in *in vitro* surrogate neutralization assays (Garaicoechea et al., 2015). Anti-rotavirus and anti-norovirus VHH would therefore appear to have a great potential as immunotherapeutic agents in infections caused by these pathogens.

#### VHH as Anti-Tumoral Agents

VHH are gaining importance as anti-tumoral agents. Their small size, tumor penetrating capacity, and homogeneous distribution compared to mAb can lead to improved treatment results, and even tumor eradication (Bannas et al., 2015). In addition, the discovery and production costs for VHH are lower than those associated with mAb, perhaps allowing for more affordable and sustainable cancer treatments. The absence in VHH of the fragment crystallizable (Fc) region of IgG, which is present in mAbs, might also reduce the adverse immune responses associated with this domain (Steeland et al., 2016). However, its absence might also reduce the efficacy of VHH in cancer treatment (Steeland et al., 2016; Hu et al., 2017) since it triggers the antibody-dependent cellular cytotoxicity (ADCC) and complement-dependent cytotoxicity (CDC) reactions upon antigen binding; both are important in tumor eradication (Weiner et al., 2010). These findings have together boosted the search for VHH anti-tumoral agents (Oliveira et al., 2013; Kijanka et al., 2015; Steeland et al., 2016; Hu et al., 2017; Iezzi et al., 2018).

The growth of certain gastrointestinal tumors has already been reported to be reduced by some VHH (Cortez-Retamozo et al., 2004; Rozan et al., 2013). VHH have been designed to elicit the ADCC and CDC responses, such as a Fab-like bispecific antibody that comprised a VHH targeting the carcinoembryonic antigen (CEA; a well-characterized tumor marker of interest for mAb-based cancer therapy) and a VHH that specifically bound and activated the FcγRIIIa receptor (an Fc-receptor class with important anti-tumoral effects) (Rozan et al., 2013). This antibody triggered potent *in vitro* cell-mediated cytotoxicity toward different CEA-positive human tumor cell lines (including human colon adenocarcinoma and primary pancreatic adenocarcinoma lines) in the presence of natural killer cells used as effector cells. Further, it showed potent *in vivo* activity, inhibiting tumor growth after being injected intraperitoneally together with human peripheral blood mononuclear cells in mice xenografted with a CEA-positive pancreatic cell line.

Other anti-tumoral VHH have been designed to block inhibitory immune checkpoints (Zhang et al., 2017). These are inhibitory pathways that under normal physiological conditions prevent autoimmunity and modulate immune response against microbial infections to prevent tissue damage (Tsai and Hsu, 2017). Some inhibitory immune checkpoints are upregulated in many cancers as a mechanism of tumor resistance to, and evasion of, the host immune system (Zou and Chen, 2008). Antibodies that block immunosuppressive checkpoints are therefore emerging as promising strategies (Pardoll, 2012; La-Beck et al., 2015; Singh et al., 2015; Shi et al., 2018). For example, immunotherapies based on the blockade of PD-1/PD-L1 (PD-1, or programmed death protein-1, is a surface cell receptor present in activated T-cells, and PD-L1, or ligand 1 of PD-1, is abundant in cells of colon cancer and other carcinomas) (Dong et al., 2002; Droeser et al., 2013) using anti-PD-1 (Lipson, 2013; Le et al., 2015, 2017; O’Neil et al., 2017) or anti-PD-L1 mAb (Herbst et al., 2014; Singh et al., 2015) have led to remarkable clinical responses in patients with colorectal cancer. In the context of VHH, Zhang et al. (2017) developed an intraperitoneally administered anti-PD-L1 nanobody fused to the Fc-domain of human IgG1 that efficiently inhibited the PD-1/PD-L1 interaction, induced the T-cell cytokine production *in vitro*, and caused the *in vivo* inhibition of tumor growth in a melanoma xenograft mouse model.

Another immunotherapeutic strategy aimed at impeding the growth of gastrointestinal and other tumors is the use of antibodies targeting angiogenic factors. Angiogenesis, a process whereby new blood vessels are formed from existing vessels, is over-induced in tumors. The new vessels that form in the vicinity of the tumor provide it with nutrients and oxygen (Kong et al., 2017). VHH that block overproduced pro-angiogenic factors have been shown effective in preventing tumor angiogenesis and therefore offer a promise as anti-tumoral agents (Arezumand et al., 2017). VHH have been developed that target key regulators of tumor angiogenesis, such as vascular endothelial growth factor (VEGF) (Farajpour et al., 2014; Ebrahimizadeh et al., 2015; Kazemi-Lomedasht et al., 2015), placental growth factor (PLGF) (Arezumand et al., 2016), and vascular endothelial growth factor receptor 2 (VEGFR-2) (Behdani et al., 2012; Ghavamipour et al., 2014; Ma et al., 2016), among others (Arezumand et al., 2017). In addition, they have been directed against hepatocyte growth factor (HGF) and its receptor c-Met since HGF/c-Met-signaling is involved in angiogenesis and the progression of different gastrointestinal cancers, including colon and gastric cancers (Birchmeier et al., 2003; Kijanka et al., 2015; Arezumand et al., 2017). A VHH targeting HGF reduced the growth of solid tumors in mice bearing glioblastoma xenografts (interestingly, some mice were even cured) (Vosjan et al., 2012), while an anti-c-Met VHH efficiently inhibited cell proliferation, adhesion, and *in vitro* migration in HGF-mediated multiple myeloma (Slordahl et al., 2013). VHH that target angiogenic factors may therefore have a bright future as anti-tumoral/anti-metastatic immunotherapeutic agents.

## Lab that Produce VHH

The small size and the hydrophilic and single-domain nature of VHH allow for their easy production in different eukaryotic systems, including yeasts (e.g., *Saccharomyces cerevisiae* and *Pichia pastoris*), fungi (e.g., *Aspergillus awamori* and *Aspergillus oryzae*), plants (e.g., *Nicotiana benthamiana* and *Arabidopsis thaliana*), mammalian cell lines [e.g., Chinese hamster ovary (CHO) cells and mouse B cells], and insect cells (e.g., those of *Spodoptera frugiperda* and *Trichoplusia ni*). They can also be produced in prokaryotic microorganisms, including Gram-negative bacteria such as *E. coli*, Gram-positive bacteria such as *Brevibacillus choshinensis,* some species of LAB, and species belonging to the genus *Bifidobacterium* (Harmsen and De Haard, 2007; Liu and Huang, 2018). The eukaryotic systems allow for the production of soluble, functional, and N-glycosylated VHH, although degradation by proteases and inefficient secretion can reduce the yield obtained. It should be remembered, however, that some of these systems, for example, mammalian cells, are quite expensive for use in large-scale production (Liu and Huang, 2018). In addition, they cannot be used for the *in situ* delivery of the VHH produced. Prokaryotic systems are therefore preferable. These can be genetically manipulated with relative ease and are more economically viable as production systems. *E. coli* is one of the microbial hosts most widely used for VHH production (Liu and Huang, 2018). However, it has some limitations. The reducing environment of its cytoplasm, for example, drives the incorrect folding of VHH and the formation of insoluble and non-functional aggregates (Liu and Huang, 2018). In addition, the presence of LPS in the bacterial membrane – which is pyrogenic in humans and other mammals (Mamat et al., 2015) – limits its use as a host.

In contrast, Gram-positive bacteria, including the LAB, have no LPS in their cell membranes (Malanovic and Lohner, 2016). This, plus the other characteristics of LAB mentioned earlier, renders this group a better alternative when producing VHH for therapeutic use. This is particularly true when they are to be orally administered as live systems for delivering VHH to the GIT (Pant et al., 2006, 2011; Vandenbroucke et al., 2010; Martin et al., 2011; Alvarez et al., 2015; Andersen et al., 2015).

*L. lactis* is one of LAB species of choice for such a delivery system. This non-pathogenic bacterium can be safely consumed; indeed, it has been used for centuries in food fermentation and preservation without raising any safety concerns, and it has been granted QPS status by the EFSA (Ricci et al., 2018) and GRAS status by the FDA (Wells and Mercenier, 2008). In addition, it is a non-invasive, non-colonizing bacterium (Steidler et al., 2000), and many genetic engineering tools are available that can induce its production of a wide range of heterologous proteins (Song et al., 2017). These advantages make *L. lactis* a successful microbial cell factory and have prompted its use as a vehicle for delivering therapeutic molecules directly into the GIT (Song et al., 2017).

Other LAB – specifically the members of the genus *Lactobacillus* – have been engineered to produce VHH directly in the GIT. These have extra advantages as VHH delivery vehicles. In addition to being safe for consumption, many *Lactobacillus* strains are normal constituents of the human gut microbiota and can survive passage to the intestine since they are resistant to gastric and bile acids. They also transiently adhere to the mucosal epithelium and have intrinsic immunostimulatory properties (Alvarez-Sieiro et al., 2014; del Rio et al., 2014). Moreover, some *Lactobacillus* strains have probiotic characteristics and are beneficial to health (Azad et al., 2018). For instance, *L. rhamnosus* GG, *L. casei* Shirota YIT9029, and *L. casei* DN-114001, among others, have antimicrobial effects against gastric and enteric bacterial pathogens and rotavirus (Lievin-Le Moal and Servin, 2014). Moreover, many genetic engineering tools, such as plasmid-based cloning systems, are available to help generate recombinant *Lactobacillus* strains capable of producing heterologous proteins (Landete, 2017). Other technologies conferring selective advantages over the use of plasmids are also available, such as genome editing for the stable integration of expression cassettes in the bacterial chromosome (based on the integrative machinery of bacteriophages) (Martin et al.1, 2000, 2011; Lin et al., 2013; Alvarez-Sieiro et al., 2014), systems based on integration by double recombination (Gosalbes et al., 2000; Steidler et al., 2003; Douglas and Klaenhammer, 2011), and technologies based on clustered regularly interspaced short palindromic repeats (CRISPR) and associated (Cas) proteins (CRISPR/Cas) (Hidalgo-Cantabrana et al., 2017; van Pijkeren and Barrangou, 2017). *Lactobacillus* is currently regarded as the most suitable LAB genus for the oral delivery of VHH against diseases of the GIT. Indeed, *Lactobacillus paracasei* was the first LAB species engineered to produce VHH, which were termed as “lactobodies” (Pant et al., 2006).

The following lines provide a comprehensive review of all the recombinant LAB that have been genetically engineered to deliver VHH antibodies *in situ* at the GIT mucosa.

### LAB That Produce VHH Against Gastrointestinal Inflammatory Diseases

**Figure 1:**
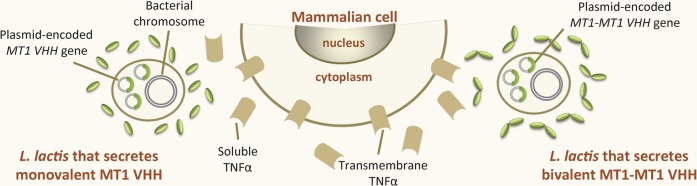
Recombinant LAB that produce VHH against intestinal inflammatory bowel diseases. Recombinant *Lactococcus lactis* strains secreting either monovalent MT1 or bivalent MT1-MT1 VHH against TNFα, an inflammatory cytokine involved in inflammatory bowel disease. The gene encoding MT1 VHH is constitutively expressed within a replicative plasmid either as a single copy (monovalent form, MT1 VHH) or as two copies in tandem (bivalent form, MTI-MT1 VHH).

Tumor necrosis factor alpha (TNFα) is one of the major cytokines involved in the pathogenesis of IBD (Billmeier et al., 2016), and immunotherapy with anti-TNFα antibodies is becoming recognized as an efficient treatment (Billmeier et al., 2016). Vandenbroucke et al. (2010) developed recombinant *L. lactis* strains that secreted monovalent (MT1) and bivalent (MT1-MT1; constructed using two MT1 and a llama IgG2a upper hinge) (Coppieters et al., 2006) VHH against murine TNFα (Figure 1). Both the MT1 and (especially) the MT1-MT1 VHH neutralized the soluble and the transmembrane forms of TNFα *in vitro*. The same study revealed the *in vivo* therapeutic efficacy of orally administered recombinant *L. lactis* secreting MT1-MT1 in terms of reducing the symptoms of chronic colitis in two mouse models (DSS-induced colitis and established colitis in IL-10^−/−^ mice). Importantly, the study also showed that the production of MT1-MT1 at the site of inflammation avoided the increase in the incidence of systemic viral and bacterial infections sometimes observed after the intravenous administration of purified anti-TNFα IgG1 antibodies. This result is of great importance since current treatments for Crohn’s disease and ulcerative colitis involve the intravenous administration of anti-TNFα IgG1 antibodies (Colombel et al., 2004; Zabana et al., 2010). The oral administration of anti-TNFα VHH-producing *L. lactis* provides a way of treating chronic GIT inflammation locally, avoiding the adverse effects associated with systemically administered anti-TNFα antibodies.

### LAB That Produce VHH Targeting Bacterial Toxins

Lactobacilli have been engineered to produce VHH to combat gastrointestinal infections caused by *C. difficile*, the leading cause of nosocomial infections in healthcare settings (Aktories et al., 2017), and of most antibiotic-associated diarrheal disease. It is also a causal agent of pseudomembranous colitis, megacolon, and chronic infections and can even cause death (Aktories et al., 2018). *C. difficile* produces two secreted enzymatic exotoxins TcdA and TcdB (Aktories et al., 2017); these are major virulence factors of this bacterium and are clearly responsible for associated diarrhea and colitis (Chandrasekaran and Lacy, 2017). TcdA and TcdB exert their effects by binding to surface receptors on intestinal epithelial cells (Aktories et al., 2017).

**Figure 2:**
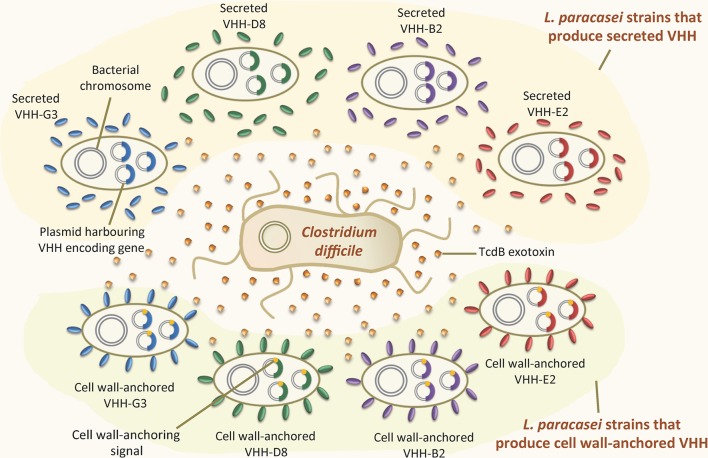
Recombinant LAB that produce VHH against the TcdB exotoxin of *Clostridium difficile.* Recombinant *Lactobacillus paracasei* strains producing four different VHH (G3, D8, B2, and E2) against the TcdB exotoxin of *Clostridium difficile*. Some of these strains secrete the VHH into the extracellular environment, while others produce it covalently attached to the cell wall. The VHH-encoding genes are constitutively expressed within replicative plasmids inside the *L. paracasei* cells.

Andersen et al. (2015) proposed an oral antitoxin strategy for the prevention and treatment of *C. difficile*-associated diarrhea, based on the neutralization of TcdB using engineered *L. paracasei* BL23 that constitutively produced anti-TcdB VHH in the GIT (Figure 2). These authors generated several recombinant *L. paracasei* strains that produced neutralizing anti-TcdB VHH that targeted different epitopes of the exotoxin. The bacteria were engineered to either secrete anti-TcdB VHH into the extracellular medium or produce them anchored to the cell wall. Both types neutralized TcdB *in vitro*. Importantly, the oral administration of a combination of two strains displaying different cell wall-anchored anti-TcdB VHH delayed the development of *C. difficile* infection and provided partial protection in a hamster protection model (Andersen et al., 2015). These results confirmed the prophylactic effect of such VHH against this pathogen. It should also be noted, however, that the oral administration of purified anti-TcdB VHH produced by a yeast provided no such protection *in vivo*, probably due to the proteolytic degradation of the antibodies under the harsh conditions of the GIT (Vandenbroucke et al., 2010). These results highlight the advantages of the continuous production of neutralizing VHH anchored to the cell wall and acting *in situ* in the GIT: this system avoids the degradation suffered by purified VHH and might prevent the diffusion of the toxin by sequestering it on the bacterial surface. Engineered lactobacilli producing surface-exposed anti-TcdB VHH might be used to complement existing therapies designed to tackle *C. difficile* infection.

### LAB That Produce VHH Against Viruses

**Figure 3:**
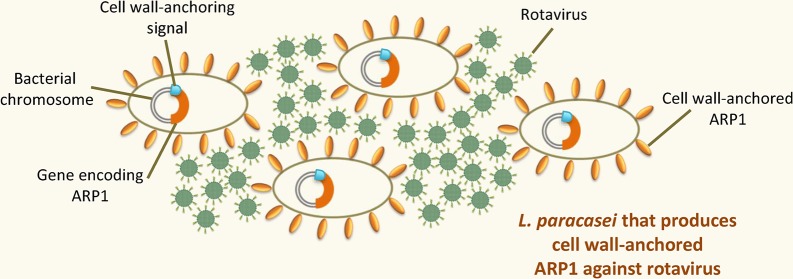
Recombinant LAB that produce VHH against rotavirus. A recombinant *Lactobacillus paracasei* strain with ARP1, targeting rhesus-monkey rotavirus serotype G3, anchored to its cell wall. The expression cassette harboring the gene encoding ARP1 is fused to a cell wall-anchoring signal sequence and is stably integrated within the bacterial chromosome.

Most of the lactobacilli engineered to produce VHH have been developed to treat or provide protection against rotavirus infection (Figure 3). Pant et al. (2006) proposed an oral prophylactic therapy to prevent rotavirus-associated diarrhea based on engineered *Lactobacillus* that produced anti-rotavirus VHH *in situ* in the GIT. This was the first time that lactobacilli were successfully engineered to produce VHH. Using a plasmid expression system, *L. paracasei* was engineered to constitutively express secreted or cell wall-anchored VHH1 (referred to as anti-rotavirus proteins [ARP1] in the present work), a VHH antibody that targets the RRV strain of rhesus monkey rotavirus serotype G3 (RRV). The study showed that both VHH1-secreting lactobacilli and VHH1-anchored lactobacilli bound and neutralized rotavirus *in vitro* in a dose-dependent manner. Moreover, VHH1-anchored lactobacilli were able to survive and constitutively produce VHH1 antibodies *in situ* in the jejunum and ileum after being orally administered to both uninfected and RRV-infected mice. The results highlighted the better performance of VHH1-anchored lactobacilli in preventing rotavirus infection and rotavirus-associated diarrhea in a mouse pup model of rotavirus infection. The good prophylactic effect observed was probably due to the numerous anti-rotavirus VHH antibodies exposed on the bacterial surface; these likely increased affinity for the viral particles, enhanced their agglutination, and facilitated their subsequent clearance. The same study also showed that recombinant VHH1-anchored lactobacilli maintain their protective activity after they are lyophilized and reconstituted. They can therefore be stored in lyophilized format, eliminating the dependence on a cold chain for their preservation, with all the advantages this brings.

In a later study, Pant et al. (2011) showed that increasing the specificity of anti-rotavirus VHH improves the prophylactic and therapeutic effects of orally administered recombinant lactobacilli. These authors generated different recombinant *L. paracasei* strains that produced anti-rotavirus VHH with different valency formats (monovalent and bivalent VHH) and specificity (monospecific and bispecific VHH) that recognized different epitopes of the viral particle and that cross-reacted with and cross-neutralized different rotavirus serotypes *in vitro* (Pant et al., 2011; Aladin et al., 2012). These strains produced either: 1) cell wall-anchored monovalent, monospecific ARP1 (referred to as VHH1 in the original study) or anti-rotavirus protein 3 (ARP3), 2) bivalent, monospecific ARP1-ARP1, ARP3-ARP3, or 3) bivalent, bispecific ARP1-ARP3 proteins. Although all the strains producing anchored VHH bound to the RRV strain *in vitro*, the bispecific ARP1-ARP3 format returned the best results. In addition, the latter neutralized RRV *in vitro* and reduced the infection rate. This strain showed the greatest *in vivo* prophylactic effect after being orally administered in a mouse pup model of rotavirus infection. In addition, in therapeutic use, it most strongly reduced the prevalence, severity, and duration of diarrheal disease. The generation of recombinant lactobacilli producing multivalent and multispecific VHH against rotavirus might improve their prophylactic and therapeutic efficacy against a broad range of viral serotypes.

More recently, Gunaydin et al. (2014) developed several expression cassettes that were cloned in tandem to allow the co-expression of ARP1 and ARP3 as either secreted or cell wall-anchored forms in *L. paracasei* BL23. Using these cassettes, they generated three recombinant strains: *L. paracasei* pAF1300 producing secreted ARP1 and anchored ARP3, *L. paracasei* pAF1400 producing anchored ARP1 and ARP3, and *L. paracasei* pAF1200 producing secreted ARP1 and ARP3. Those producing ARP1 and ARP3 in anchored form bound a broad range of human and simian rotavirus serotypes with great affinity. Secreted ARP1 produced by *L. paracasei* pAF1300 and *L. paracasei* pAF1200 and secreted ARP3 produced by *L. paracasei* pAF1200 bound to different human rotavirus serotypes and simian rotavirus RRV. Analysis of the mechanism of interaction between rotavirus and *L. paracasei* pAF1300 suggested that once the virus is captured by anchored ARP3, the bacterium produces secreted ARP1, which targets other rotavirus epitopes. This confirms the feasibility of engineering *Lactobacillus* to co-produce VHH with different specificities and cell locations, increasing the chances of neutralizing different rotavirus serotypes and reducing the chances of escape mutants appearing. Moreover, the simultaneous production of several VHH with different specificities in one engineered *Lactobacillus* would considerably reduce production costs compared to preparing cocktails of different recombinant lactobacilli, each producing different VHH.

The recombinant lactobacilli described in the above-mentioned studies relied on plasmid expression systems for the production and delivery of anti-rotavirus VHH. These systems have been widely used to overproduce heterologous proteins in LAB, but they suffer drawbacks that hamper their use as delivery systems for the *in situ* production of therapeutic proteins in the GIT. For instance, expression plasmids show structural and segregational instability, and their maintenance in cells therefore requires a selection pressure, commonly resistance to antibiotics. The presence of antibiotic resistance genes in therapeutic recombinant lactobacilli, however, is undesirable since antibiotics would be needed to select VHH-producing strains in the intestine. In addition, they would increase the risk of antibiotic resistance genes being horizontally transferred to other, perhaps pathogenic, GIT bacteria.

Given the above, different approaches have been proposed to maintain heterologous genes in recombinant LAB. A feasible alternative is the use of food-grade expression vectors with selection markers other than antibiotics that provide new phenotypes (e.g., immunity to bacteriocins, utilization of rare sugars, etc.) or complement specific mutations that restore impaired functions (e.g., complementation of auxotrophic mutants depending on threonine, thymidine, alanine, lactose, etc.) (Landete, 2017). But in any case, it is necessary to maintain a selective pressure in the GIT, which can be avoided by the chromosomal integration of the heterologous genes. Martin et al. (2011) developed a universal integrative expression system that allows the stable integration of expression cassettes for the production of scFv and VHH in the chromosome of *Lactobacillus*. This system is based on an ingenious genome editing technology, developed by Martin et al. (2000), that involves the site-specific integration of heterologous genes into the LAB chromosome, plus a purification system that – once the vector is integrated into the chromosome – leads to the elimination of all non-food-grade deoxyribonucleic acid, including the antibiotic selection genes. This system allowed the generation of different food-grade recombinant *L. paracasei* strains that stably produce VHH against rotavirus (Martin et al., 2011). The recombinant *L. paracasei* EM233 strain, which stably produces cell wall-anchored ARP1 (Figure 3), was shown to effectively bind rotavirus *in vitro*, and importantly, the integrated gene encoding APR1 VHH antibody was stable with no need for any selective pressure. After its oral administration, the recombinant *L. paracasei* strain producing anchored ARP1 showed prophylactic efficacy, reducing the duration and severity of diarrhea in a mouse model of rotavirus infection. More recently, *L. rhamnosus* GG was genetically engineered using this system to stably produce cell wall-anchored ARP1 antibody against rotavirus (Alvarez et al., 2015). *L. rhamnosus* GG is a well-known probiotic strain that, in addition to having intrinsic anti-rotavirus activity (Lievin-Le Moal and Servin, 2014; Guarino et al., 2015), helps reinforce the intestinal barrier, triggers the innate and adaptive immune responses (Sindhu et al., 2014), and transiently colonizes the intestine (Alander et al., 1999). It is therefore an ideal vehicle for the production of VHH against rotavirus in the GIT mucosa. Alvarez et al. (2015) tested the capacity of several *L. rhamnosus* GG strains to display anchored ARP1 on their surfaces for which they used the plasmid-based expression system described above (Martin et al., 2011). Although all these strains produced ARP1, only one, a naturally occurring exopolysaccharide-deficient mutant strain named *L. rhamnosus* GG (UT), efficiently produced anchored ARP1. Genomic analysis suggested that two genes (*welE* and *welF*) belonging to the exopolysaccharide (EPS) cluster were inactive in this strain, which impaired the production of cell-bound EPS. The EPS deficiency allowed the appearance of the anchored ARP1; in all the other strains tested the thick layer of EPS masked it. *L. rhamnosus* GG (UT) was further genetically engineered to produce ARP1 anchored to the cell wall through the use of the above-described integrative-expression system (Martin et al., 2011). This strain, known as *L. rhamnosus* GG EM233, bounded rotavirus particles and seemed to confer a protective effect *in vivo*; when it was orally administered to mice before they were infected with rotavirus, the prevalence, duration, and severity of diarrhea were reduced compared to the control group (Alvarez et al., 2015).

Thus, the oral administration of engineered lactobacilli that either secrete VHH or display cell wall-anchored VHH (with single or multiple valencies and specificities) may be a way of preventing or treating rotavirus infection and its associated diarrhea. The site-specific integration expression technology used in the above studies represents an important advance in the genetic manipulation of LAB for the generation of food-grade, live recombinant bacteria that produce prophylactic and therapeutic VHH *in situ* in the GIT.

## Concluding Remarks and Future Perspectives

Over the last decade, LAB have emerged as powerful oral delivery vectors for the *in situ* production of VHH at the GIT mucosa where they may act as therapeutic agents or provide passive protection against different gastrointestinal diseases. Specific LAB strains have been genetically modified to either secrete VHH or produce them anchored to their surfaces. This has been achieved using expression systems based on replicative plasmids, which require a selection pressure such as the presence of antibiotic resistance genes for their maintenance in bacteria (Pant et al., 2006, 2011; Vandenbroucke et al., 2010; Gunaydin et al., 2014; Andersen et al., 2015) or by using other genetic tools that allow the integration of VHH-encoding genes into the bacterial chromosome. This provides stability to the integrated gene without the need for a selection pressure system (Martin et al., 2011; Alvarez et al., 2015). In the near future, other powerful chromosome editing technologies, such as those based on the CRISPR/Cas systems (Hidalgo-Cantabrana et al., 2017), might also be used to generate recombinant LAB that stably produce VHH against diseases of the GIT. At the time of writing, only *L. lactis* (Vandenbroucke et al., 2010), *L. paracasei* (Pant et al., 2006, 2011; Martin et al., 2011; Gunaydin et al., 2014; Andersen et al., 2015), and *L. rhamnosus* (Alvarez et al., 2015) have been engineered to produce VHH for treating or preventing GIT disease. Many LAB strains have been described as possessing probiotic characteristics of benefit against IBD (Saez-Lara et al., 2015), and against the bacteria (Goderska et al., 2018; Lin et al., 2018; McFarland et al., 2018) and viruses (Guarino et al., 2015) that cause gastrointestinal infections. Some of them even show anti-tumoral properties against colorectal cancer (Ambalam et al., 2016). Based on their intrinsic benefits, specific probiotic LAB strains could be selected and genetically modified to produce therapeutic or prophylactic VHH. This would result in a synergistic effect against the target disease. So far, recombinant LAB that produce VHH have been directed to prevent or treat IBD (Vandenbroucke et al., 2010) and infections caused by *C. difficile* (Andersen et al., 2015) and rotavirus (Pant et al., 2006, 2011; Martin et al., 2011; Gunaydin et al., 2014; Alvarez et al., 2015). Given the great potential of VHH as therapeutic and prophylactic agents, many other LAB might be engineered to produce VHH targeting other diseases of the GIT, such as infections caused by pathogenic bacteria (e.g., *C. jejuni*, *H. pylori*, ETEC, STEC, *Salmonella*, etc.), viruses (e.g., norovirus), fungi (e.g., *C. albicans*), and intestinal parasites and to provide relief from food allergies and intolerances, inflammatory diseases, and even gastrointestinal cancers. They might also be produced to treat disease in other mucous membranes inhabited by LAB.

## Data Availability

No datasets were generated or analyzed for this study.

## Author Contributions

All authors listed have made a substantial, direct and intellectual contribution to the work, and approved it for publication.

### Conflict of Interest Statement

The authors declare that the research was conducted in the absence of any commercial or financial relationships that could be construed as a potential conflict of interest.

## References

[ref1] Abbady A. Q. Al-Daoude A. Al-Mariri A. Zarkawi M. Muyldermans S. (2012). Chaperonin GroEL a Brucella immunodominant antigen identified using nanobody and MALDI-TOF-MS technologies. Vet. Immunol. Immunopathol. 146**,** 254–263. 10.1016/j.vetimm.2012.01.015, PMID: 22472910

[ref2] Adel-Patient K. Ah-Leung S. Creminon C. Nouaille S. Chatel J. M. Langella P.. (2005). Oral administration of recombinant *Lactococcus lactis* expressing bovine beta-lactoglobulin partially prevents mice from sensitization. Clin. Exp. Allergy 35, 539–546. 10.1111/j.1365-2222.2005.02225.x, PMID: 15836765

[ref3] Ahmed B. Loos M. Vanrompay D. Cox E. (2014). Oral immunization with *Lactococcus lactis*-expressing EspB induces protective immune responses against *Escherichia coli* O157:H7 in a murine model of colonization. Vaccine 32, 3909–3916. 10.1016/j.vaccine.2014.05.054, PMID: 24877767

[ref4] Aktories K. Papatheodorou P. Schwan C. (2018). Binary *Clostridium difficile* toxin (CDT)—A virulence factor disturbing the cytoskeleton. Anaerobe 53, 21–29. 10.1016/j.anaerobe.2018.03.001, PMID: 29524654

[ref5] Aktories K. Schwan C. Jank T. (2017). Clostridium difficile toxin biology. Annu. Rev. Microbiol. 71**,** 281–307. 10.1146/annurev-micro-090816-093458, PMID: 28657883

[ref6] Aladin F. Einerhand A. W. Bouma J. Bezemer S. Hermans P. Wolvers D. (2012). *In vitro* neutralisation of rotavirus infection by two broadly specific recombinant monovalent llama-derived antibody fragments. PLoS One 7:e32949. 10.1371/journal.pone.0032949 22403728 PMC3293919

[ref7] Alander M. Satokari R. Korpela R. Saxelin M. Vilpponen-Salmela T. Mattila-Sandholm T.. (1999). Persistence of colonization of human colonic mucosa by a probiotic strain, *Lactobacillus rhamnosus* GG, after oral consumption. Appl. Environ. Microbiol. 65, 351–354., PMID: 9872808 10.1128/aem.65.1.351-354.1999 PMC91031

[ref8] Alvarez B. Krogh-Andersen K. Tellgren-Roth C. Martinez N. Gunaydin G. Lin Y.. (2015). An exopolysaccharide-deficient mutant of *Lactobacillus rhamnosus* GG efficiently displays a protective llama antibody fragment against rotavirus on its surface. Appl. Environ. Microbiol. 81**,** 5784–5793. 10.1128/aem.00945-15, PMID: 26092449 PMC4551240

[ref9] Alvarez-Sieiro P. Martin M. C. Redruello B. del Rio B. Ladero V. Palanski B. A.. (2014). Generation of food-grade recombinant *Lactobacillus casei* delivering *Myxococcus xanthus* prolyl endopeptidase. Appl. Microbiol. Biotechnol. 98, 6689–6700. 10.1007/s00253-014-5730-7, PMID: 24752841 PMC4393947

[ref10] Ambalam P. Raman M. Purama R. K. Doble M. (2016). Probiotics, prebiotics and colorectal cancer prevention. Best Pract. Res. Clin. Gastroenterol. 30, 119–131. 10.1016/j.bpg.2016.02.009, PMID: 27048903

[ref11] Andersen K. K. Marcotte H. Alvarez B. Boyaka P. N. Hammarstrom L. (2011). *In situ* gastrointestinal protection against anthrax edema toxin by single-chain antibody fragment producing lactobacilli. BMC Biotechnol. 11:126. 10.1186/1472-6750-11-126, PMID: 22185669 PMC3295704

[ref12] Andersen K. K. Strokappe N. M. Hultberg A. Truusalu K. Smidt I. Mikelsaar R. H. (2015). Neutralization of *Clostridium difficile* toxin B mediated by engineered lactobacilli producing single domain antibodies. Infect. Immun. 84, 395–406. 10.1128/iai.00870-15 26573738 PMC4730582

[ref13] Ardekani L. S. Gargari S. L. Rasooli I. Bazl M. R. Mohammadi M. Ebrahimizadeh W.. (2013). A novel nanobody against urease activity of *Helicobacter pylori*. Int. J. Infect. Dis. 17**,** e723–728. 10.1016/j.ijid.2013.02.015, PMID: 23561799

[ref14] Arezumand R. Alibakhshi A. Ranjbari J. Ramazani A. Muyldermans S. (2017). Nanobodies as novel agents for targeting angiogenesis in solid cancers. Front. Immunol. 8:1746. 10.3389/fimmu.2017.01746 29276515 PMC5727022

[ref15] Arezumand R. Mahdian R. Zeinali S. Hassanzadeh-Ghassabeh G. Mansouri K. Khanahmad H.. (2016). Identification and characterization of a novel nanobody against human placental growth factor to modulate angiogenesis. Mol. Immunol. 78**,** 183–192. 10.1016/j.molimm.2016.09.012, PMID: 27648860

[ref16] Azad M. A. K. Sarker M. Li T. Yin J. (2018). Probiotic species in the modulation of gut microbiota: an overview. Biomed. Res. Int. 2018:9478630. 10.1155/2018/9478630, PMID: 29854813 PMC5964481

[ref17] Bakherad H. Mousavi Gargari S. L. Rasooli I. Rajabibazl M. Mohammadi M. Ebrahimizadeh W.. (2013). *In vivo* neutralization of botulinum neurotoxins serotype E with heavy-chain camelid antibodies (VHH). Mol. Biotechnol. 55**,** 159–167. 10.1007/s12033-013-9669-1, PMID: 23666874

[ref18] Bannas P. Lenz A. Kunick V. Well L. Fumey W. Rissiek B.. (2015). Molecular imaging of tumors with nanobodies and antibodies: timing and dosage are crucial factors for improved *in vivo* detection. Contrast Media Mol. Imaging 10**,** 367–378. 10.1002/cmmi.1637, PMID: 25925493

[ref19] Baral T. N. Magez S. Stijlemans B. Conrath K. Vanhollebeke B. Pays E.. (2006). Experimental therapy of African trypanosomiasis with a nanobody-conjugated human trypanolytic factor. Nat. Med. 12**,** 580–584. 10.1038/nm1395 , PMID: 16604085

[ref20] Behdani M. Zeinali S. Khanahmad H. Karimipour M. Asadzadeh N. Azadmanesh K.. (2012). Generation and characterization of a functional nanobody against the vascular endothelial growth factor receptor-2; angiogenesis cell receptor. Mol. Immunol. 50**,** 35–41. 10.1016/j.molimm.2011.11.013, PMID: 22208996

[ref21] Behnsen J. Deriu E. Sassone-Corsi M. Raffatellu M. (2013). Probiotics: properties, examples, and specific applications. Cold Spring Harb. Perspect. Med. 3:a010074. 10.1101/cshperspect.a010074, PMID: 23457295 PMC3579206

[ref22] Benbouziane B. Ribelles P. Aubry C. Martin R. Kharrat P. Riazi A.. (2013). Development of a stress-inducible controlled expression (SICE) system in *Lactococcus lactis* for the production and delivery of therapeutic molecules at mucosal surfaces. J. Biotechnol. 168**,** 120–129. 10.1016/j.jbiotec.2013.04.019, PMID: 23664884

[ref23] Bermudez-Humaran L. G. Kharrat P. Chatel J. M. Langella P. (2011). Lactococci and lactobacilli as mucosal delivery vectors for therapeutic proteins and DNA vaccines. Microb. Cell Fact. 10 (Suppl 1):S4. 10.1186/1475-2859-10-S1-S4, PMID: 21995317 PMC3231930

[ref24] Bermudez-Humaran L. G. Motta J. P. Aubry C. Kharrat P. Rous-Martin L. Sallenave J. M. (2015). Serine protease inhibitors protect better than IL-10 and TGF-beta anti-inflammatory cytokines against mouse colitis when delivered by recombinant lactococci. Microb. Cell Fact. 14:26. 10.1186/s12934-015-0198-4 25889561 PMC4371826

[ref25] Bernardeau M. Vernoux J. P. Henri-Dubernet S. Gueguen M. (2008). Safety assessment of dairy microorganisms: the *Lactobacillus* genus. Int. J. Food Microbiol. 126, 278–285. 10.1016/j.ijfoodmicro.2007.08.015, PMID: 17889388

[ref26] Billmeier U. Dieterich W. Neurath M. F. Atreya R. (2016). Molecular mechanism of action of anti-tumor necrosis factor antibodies in inflammatory bowel diseases. World J. Gastroenterol. 22**,** 9300–9313. 10.3748/wjg.v22.i42.9300, PMID: 27895418 PMC5107694

[ref27] Birchmeier C. Birchmeier W. Gherardi E. Vande Woude G. F. (2003). Met, metastasis, motility and more. Nat. Rev. Mol. Cell Biol. 4**,** 915–925. 10.1038/nrm1261, PMID: 14685170

[ref28] Braat H. Rottiers P. Hommes D. W. Huyghebaert N. Remaut E. Remon J. P.. (2006). A phase I trial with transgenic bacteria expressing interleukin-10 in Crohn’s disease. Clin. Gastroenterol. Hepatol. 4**,** 754–759. 10.1016/j.cgh.2006.03.028, PMID: 16716759

[ref29] Bradley M. E. Dombrecht B. Manini J. Willis J. Vlerick D. De Taeye S. (2015). Potent and efficacious inhibition of CXCR2 signaling by biparatopic nanobodies combining two distinct modes of action. Mol. Pharmacol. 87**,** 251–262. 10.1124/mol.114.094821 25468882

[ref30] Cano-Garrido O. Seras-Franzoso J. Garcia-Fruitos E. (2015). Lactic acid bacteria: reviewing the potential of a promising delivery live vector for biomedical purposes. Microb. Cell Fact. 14:137. 10.1186/s12934-015-0313-6 26377321 PMC4573465

[ref31] Chancey C. J. Khanna K. V. Seegers J. F. Zhang G. W. Hildreth J. Langan A.. (2006). *Lactobacilli*-expressed single-chain variable fragment (scFv) specific for intercellular adhesion molecule 1 (ICAM-1) blocks cell-associated HIV-1 transmission across a cervical epithelial monolayer. J. Immunol. 176**,** 5627–5636. 10.4049/jimmunol.176.9.5627, PMID: 16622032

[ref32] Chandrasekaran R. Lacy D. B. (2017). The role of toxins in *Clostridium difficile* infection. FEMS Microbiol. Rev. 41**,** 723–750. 10.1093/femsre/fux048, PMID: 29048477 PMC5812492

[ref33] Colombel J. F. Loftus E. V. Jr. Tremaine W. J. Egan L. J. Harmsen W. S. Schleck C. D.. (2004). The safety profile of infliximab in patients with Crohn’s disease: the Mayo clinic experience in 500 patients. Gastroenterology 126**,** 19–31. 10.1053/j.gastro.2003.10.047, PMID: 14699483

[ref34] Coppieters K. Dreier T. Silence K. de Haard H. Lauwereys M. Casteels P.. (2006). Formatted anti-tumor necrosis factor alpha VHH proteins derived from camelids show superior potency and targeting to inflamed joints in a murine model of collagen-induced arthritis. Arthritis Rheum. 54**,** 1856–1866. 10.1002/art.21827 , PMID: 16736523

[ref35] Cortes-Perez N. G. Bermudez-Humaran L. G. Le Loir Y. Rodriguez-Padilla C. Gruss A. Saucedo-Cardenas O.. (2003). Mice immunization with live lactococci displaying a surface anchored HPV-16 E7 oncoprotein. FEMS Microbiol. Lett. 229, 37–42. 10.1016/S0378-1097(03)00778-X, PMID: 14659540

[ref36] Cortez-Retamozo V. Backmann N. Senter P. D. Wernery U. De Baetselier P. Muyldermans S.. (2004). Efficient cancer therapy with a nanobody-based conjugate. Cancer Res. 64**,** 2853–2857. 10.1158/0008-5472.CAN-03-3935, PMID: 15087403

[ref37] Cortez-Retamozo V. Lauwereys M. Hassanzadeh Gh G. Gobert M. Conrath K. Muyldermans S.. (2002). Efficient tumor targeting by single-domain antibody fragments of camels. Int. J. Cancer 98, 456–462. 10.1002/ijc.10212, PMID: 11920600

[ref38] Daniel C. Roussel Y. Kleerebezem M. Pot B. (2011). Recombinant lactic acid bacteria as mucosal biotherapeutic agents. Trends Biotechnol. 29**,** 499–508. 10.1016/j.tibtech.2011.05.002, PMID: 21665301

[ref39] De Meyer T. Muyldermans S. Depicker A. (2014). Nanobody-based products as research and diagnostic tools. Trends Biotechnol. 32**,** 263–270. 10.1016/j.tibtech.2014.03.001, PMID: 24698358

[ref40] de Ruyter P. G. Kuipers O. P. de Vos W. M. (1996). Controlled gene expression systems for *Lactococcus lactis* with the food-grade inducer nisin. Appl. Environ. Microbiol. 62**,** 3662–3667. PMID: 8837421 10.1128/aem.62.10.3662-3667.1996 PMC168174

[ref41] del Rio B. Dattwyler R. J. Aroso M. Neves V. Meirelles L. Seegers J. F.. (2008). Oral immunization with recombinant *Lactobacillus plantarum* induces a protective immune response in mice with Lyme disease. Clin. Vaccine Immunol. 15**,** 1429–1435. 10.1128/CVI.00169-08, PMID: 18632920 PMC2546682

[ref42] del Rio B. Fuente J. L. Neves V. Dattwyler R. Seegers J. F. Gomes-Solecki M. (2010). Platform technology to deliver prophylactic molecules orally: an example using the Class A select agent *Yersinia pestis*. Vaccine 28, 6714–6722. 10.1016/j.vaccine.2010.07.084 20699130 PMC2942074

[ref43] del Rio B. Martín M. C. Sarmiento M. E. Acosta A. Alvarez M. A. (2014). “Lactobacilli as live vectors for oral vaccination against TB,” in The art and science of tuberculosis vaccine development. eds. Norazmi M. N. Acosta A. Sarmiento M. E. (Shah Alam, Selangor, Malaysia: Oxford University Press), 794–811.

[ref44] Desvaux M. Dumas E. Chafsey I. Hebraud M. (2006). Protein cell surface display in Gram-positive bacteria: from single protein to macromolecular protein structure. FEMS Microbiol. Lett. 256, 1–15. 10.1111/j.1574-6968.2006.00122.x , PMID: 16487313

[ref45] Dieye Y. Usai S. Clier F. Gruss A. Piard J. C. (2001). Design of a protein-targeting system for lactic acid bacteria. J. Bacteriol. 183, 4157–4166. 10.1128/jb.183.14.4157-4166.2001, PMID: 11418555 PMC95304

[ref46] Ding C. Ma J. Dong Q. Liu Q. (2018). Live bacterial vaccine vector and delivery strategies of heterologous antigen: a review. Immunol. Lett. 197, 70–77. 10.1016/j.imlet.2018.03.006, PMID: 29550258

[ref47] Dong H. Strome S. E. Salomao D. R. Tamura H. Hirano F. Flies D. B.. (2002). Tumor-associated B7-H1 promotes T-cell apoptosis: a potential mechanism of immune evasion. Nat. Med. 8**,** 793–800. 10.1038/nm730, PMID: 12091876

[ref48] Dong J. Thompson A. A. Fan Y. Lou J. Conrad F. Ho M. (2010). A single-domain llama antibody potently inhibits the enzymatic activity of botulinum neurotoxin by binding to the non-catalytic alpha-exosite binding region. J. Mol. Biol. 397, 1106–1118. 10.1016/j.jmb.2010.01.070 20138889 PMC2903050

[ref49] Douglas G. L. Klaenhammer T. R. (2011). Directed chromosomal integration and expression of the reporter gene gusA3 in *Lactobacillus acidophilus* NCFM. Appl. Environ. Microbiol. 77, 7365–7371. 10.1128/aem.06028-11, PMID: 21873486 PMC3194874

[ref50] Droeser R. A. Hirt C. Viehl C. T. Frey D. M. Nebiker C. Huber X.. (2013). Clinical impact of programmed cell death ligand 1 expression in colorectal cancer. Eur. J. Cancer 49, 2233–2242. 10.1016/j.ejca.2013.02.015, PMID: 23478000

[ref51] Dumoulin M. Conrath K. Van Meirhaeghe A. Meersman F. Heremans K. Frenken L. G.. (2002). Single-domain antibody fragments with high conformational stability. Protein Sci. 11, 500–515. 10.1110/ps.34602, PMID: 11847273 PMC2373476

[ref52] Ebrahimizadeh W. Mousavi Gargari S. L. Javidan Z. Rajabibazl M. (2015). Production of novel VHH nanobody inhibiting angiogenesis by targeting binding site of VEGF. Appl. Biochem. Biotechnol. 176, 1985–1995. 10.1007/s12010-015-1695-y, PMID: 26054617

[ref53] Eden T. Menzel S. Wesolowski J. Bergmann P. Nissen M. Dubberke G. (2018). A cDNA immunization strategy to generate nanobodies against membrane proteins in native conformation. Front. Immunol. 8:1989. 10.3389/fimmu.2017.01989 29410663 PMC5787055

[ref54] Els Conrath K. Lauwereys M. Wyns L. Muyldermans S. (2001). Camel single-domain antibodies as modular building units in bispecific and bivalent antibody constructs. J. Biol. Chem. 276**,** 7346–7350. 10.1074/jbc.M007734200, PMID: 11053416

[ref55] FAO/WHO. (2006). Probiotics in food. Health and nutritional properties and guidelines for evaluation. Rome: FAO Food Nutrition. PMID:

[ref56] Farajpour Z. Rahbarizadeh F. Kazemi B. Ahmadvand D. (2014). A nanobody directed to a functional epitope on VEGF, as a novel strategy for cancer treatment. Biochem. Biophys. Res. Commun. 446, 132–136. 10.1016/j.bbrc.2014.02.069, PMID: 24569074

[ref57] Foligne B. Dessein R. Marceau M. Poiret S. Chamaillard M. Pot B.. (2007). Prevention and treatment of colitis with *Lactococcus lactis* secreting the immunomodulatory Yersinia LcrV protein. Gastroenterology 133, 862–874. 10.1053/j.gastro.2007.06.018, PMID: 17678918

[ref58] Fredriksen L. Kleiveland C. R. Hult L. T. O. Lea T. Nygaard C. S. Eijsink V. G. H.. (2012). Surface display of N-terminally anchored invasin by *Lactobacillus plantarum* activates NF-κB in monocytes. Appl. Environ. Microbiol. 78, 5864–5871. 10.1128/AEM.01227-12, PMID: 22706054 PMC3406107

[ref59] Fredriksen L. Mathiesen G. Sioud M. Eijsink V. G. H. (2010). Cell wall anchoring of the 37-kilodalton oncofetal antigen by *Lactobacillus plantarum* for mucosal cancer vaccine delivery. Appl. Environ. Microbiol. 76, 7359–7362. 10.1128/AEM.01031-10, PMID: 20851975 PMC2976233

[ref60] Frieri M. Kumar K. Boutin A. (2017). Antibiotic resistance. J. Infect. Public Health 10, 369–378. 10.1016/j.jiph.2016.08.007, PMID: 27616769

[ref61] Garaicoechea L. Aguilar A. Parra G. I. Bok M. Sosnovtsev S. V. Canziani G.. (2015). Llama nanoantibodies with therapeutic potential against human norovirus diarrhea. PLoS One 10:e0133665. 10.1371/journal.pone.0133665, PMID: 26267898 PMC4534396

[ref62] Garaicoechea L. Olichon A. Marcoppido G. Wigdorovitz A. Mozgovoj M. Saif L.. (2008). Llama-derived single-chain antibody fragments directed to rotavirus VP6 protein possess broad neutralizing activity in vitro and confer protection against diarrhea in mice. J. Virol. 82, 9753–9764. 10.1128/jvi.00436-08, PMID: 18632867 PMC2546978

[ref63] García-Fruitós E. (2012). Lactic acid bacteria: a promising alternative for recombinant protein production. Microb. Cell Fact. 11, 157–157. 10.1186/1475-2859-11-157, PMID: 23234563 PMC3528458

[ref64] Gareau M. G. Sherman P. M. Walker W. A. (2010). Probiotics and the gut microbiota in intestinal health and disease. Nat. Rev. Gastroenterol. Hepatol. 7, 503–514. 10.1038/nrgastro.2010.117, PMID: 20664519 PMC4748966

[ref65] Gerritsen J. Smidt H. Rijkers G. T. de Vos W. M. (2011). Intestinal microbiota in human health and disease: the impact of probiotics. Genes Nutr. 6, 209–240. 10.1007/s12263-011-0229-7, PMID: 21617937 PMC3145058

[ref66] Ghavamipour F. Shahangian S. S. Sajedi R. H. Arab S. S. Mansouri K. Aghamaali M. R. (2014). Development of a highly-potent anti-angiogenic VEGF8-109 heterodimer by directed blocking of its VEGFR-2 binding site. FEBS J. 281, 4479–4494. 10.1111/febs.12956, PMID: 25132001

[ref67] Ghosh S. Malik Y. S. Kobayashi N. (2018). Therapeutics and immunoprophylaxis against noroviruses and rotaviruses: the past, present, and future. Curr. Drug Metab. 19, 170–191. 10.2174/1389200218666170912161449, PMID: 28901254 PMC5971199

[ref68] Giomarelli B. Maggi T. Younson J. Kelly C. Pozzi G. (2004). Expression of a functional single-chain Fv antibody on the surface of *Streptococcus gordonii*. Mol. Biotechnol. 28, 105–112. 10.1385/MB:28:2:105, PMID: 15477649

[ref69] Goderska K. Agudo Pena S. Alarcon T. (2018). *Helicobacter pylori* treatment: antibiotics or probiotics. Appl. Microbiol. Biotechnol. 102, 1–7. 10.1007/s00253-017-8535-7, PMID: 29075827 PMC5748437

[ref70] Goldman E. R. Liu J. L. Zabetakis D. Anderson G. P. (2017). Enhancing stability of camelid and shark single domain antibodies: an overview. Front. Immunol. 8:865. 10.3389/fimmu.2017.00865 28791022 PMC5524736

[ref71] Gomes-Santos A. C. de Oliveira R. P. Moreira T. G. Castro-Junior A. B. Horta B. C. Lemos L.. (2017). Hsp65-producing *Lactococcus lactis* prevents inflammatory intestinal disease in mice by IL-10- and TLR2-dependent pathways. Front. Immunol. 8:30. 10.3389/fimmu.2017.00030, PMID: 28194152 PMC5277002

[ref72] Gómez-Sebastián S. Nuñez M. C. Garaicoechea L. Alvarado C. Mozgovoj M. Lasa R.. (2012). Rotavirus A-specific single-domain antibodies produced in baculovirus-infected insect larvae are protective *in vivo*. BMC Biotechnol. 12**,** 59–59. 10.1186/1472-6750-12-59, PMID: 22953695 PMC3444942

[ref73] Gosalbes M. J. Esteban C. D. Galan J. L. Perez-Martinez G. (2000). Integrative food-grade expression system based on the lactose regulon of *Lactobacillus casei*. Appl. Environ. Microbiol. 66, 4822–4828. 10.1128/AEM.66.11.4822-4828.2000 , PMID: 11055930 PMC92386

[ref74] Gu Q. Song D. Zhu M. (2009). Oral vaccination of mice against *Helicobacter pylori* with recombinant *Lactococcus lactis* expressing urease subunit B. FEMS Immunol. Med. Microbiol. 56, 197–203. 10.1111/j.1574-695X.2009.00566.x, PMID: 19453750 PMC7110364

[ref75] Guarino A. Guandalini S. Lo Vecchio A. (2015). Probiotics for prevention and treatment of diarrhea. J. Clin. Gastroenterol. 49 (Suppl 1), S37–45. 10.1097/mcg.0000000000000349 26447963

[ref76] Gunaydin G. Alvarez B. Lin Y. Hammarstrom L. Marcotte H. (2014). Co-expression of anti-rotavirus proteins (llama VHH antibody fragments) in *Lactobacillus*: development and functionality of vectors containing two expression cassettes in tandem. PLoS One 9:e96409. 10.1371/journal.pone.0096409, PMID: 24781086 PMC4004553

[ref77] Guo S. Yan W. McDonough S. P. Lin N. Wu K. J. He H.. (2015). The recombinant *Lactococcus lactis* oral vaccine induces protection against *C. difficile* spore challenge in a mouse model. Vaccine 33, 1586–1595. 10.1016/j.vaccine.2015.02.006, PMID: 25698490

[ref78] Hamers-Casterman C. Atarhouch T. Muyldermans S. Robinson G. Hamers C. Songa E. B.. (1993). Naturally occurring antibodies devoid of light chains. Nature 363, 446–448. 10.1038/363446a0, PMID: 8502296

[ref79] Hanniffy S. Wiedermann U. Repa A. Mercenier A. Daniel C. Fioramonti J. (2004). Potential and opportunities for use of recombinant lactic acid bacteria in human health. Adv. Appl. Microbiol. 56**,** 1–64. 10.1016/s0065-2164(04)56001-x 15566975

[ref80] Hanson M. L. Hixon J. A. Li W. Felber B. K. Anver M. R. Stewart C. A.. (2014). Oral delivery of IL-27 recombinant bacteria attenuates immune colitis in mice. Gastroenterology 146, 210–221.e213. 10.1053/j.gastro.2013.09.060, PMID: 24120477 PMC3920828

[ref81] Harmsen M. M. De Haard H. J. (2007). Properties, production, and applications of camelid single-domain antibody fragments. Appl. Microbiol. Biotechnol. 77, 13–22. 10.1007/s00253-007-1142-2, PMID: 17704915 PMC2039825

[ref82] Harmsen M. M. Fijten H. P. Engel B. Dekker A. Eble P. L. (2009). Passive immunization with llama single-domain antibody fragments reduces foot-and-mouth disease transmission between pigs. Vaccine 27, 1904–1911. 10.1016/j.vaccine.2009.01.110, PMID: 19368770

[ref83] Herbst R. S. Soria J.-C. Kowanetz M. Fine G. D. Hamid O. Gordon M. S.. (2014). Predictive correlates of response to the anti-PD-L1 antibody MPDL3280A in cancer patients. Nature 515, 563–567. 10.1038/nature14011, PMID: 25428504 PMC4836193

[ref84] Hidalgo-Cantabrana C. O’Flaherty S. Barrangou R. (2017). CRISPR-based engineering of next-generation lactic acid bacteria. Curr. Opin. Microbiol. 37, 79–87. 10.1016/j.mib.2017.05.015, PMID: 28622636

[ref85] Hoang P. M. Cho S. Kim K. E. Byun S. J. Lee T. K. Lee S. (2015). Development of *Lactobacillus paracasei* harboring nucleic acid-hydrolyzing 3D8 scFv as a preventive probiotic against murine norovirus infection. Appl. Microbiol. Biotechnol. 99, 2793–2803. 10.1007/s00253-014-6257-7, PMID: 25487889

[ref86] Hols P. Slos P. Dutot P. Reymund J. Chabot P. Delplace B. (1997). Efficient secretion of the model antigen M6-gp41E in *Lactobacillus plantarum* NCIMB 8826. Microbiologica 143, 2733–2741. 10.1099/00221287-143-8-2733 9274026

[ref87] Hongying F. Xianbo W. Fang Y. Yang B. Beiguo L. (2014). Oral immunization with recombinant *Lactobacillus acidophilus* expressing the adhesin Hp0410 of *Helicobacter pylori* induces mucosal and systemic immune responses. Clin. Vaccine Immunol. 21, 126–132. 10.1128/cvi.00434-13, PMID: 24285819 PMC3910932

[ref88] Hoseinpoor R. Mousavi Gargari S. L. Rasooli I. Rajabibazl M. Shahi B. (2014). Functional mutations in and characterization of VHH against *Helicobacter pylori* urease. Appl. Biochem. Biotechnol. 172, 3079–3091. 10.1007/s12010-014-0750-4, PMID: 24492955

[ref89] Hosseinidoust Z. Mostaghaci B. Yasa O. Park B.-W. Singh A. V. Sitti M. (2016). Bioengineered and biohybrid bacteria-based systems for drug delivery. Adv. Drug Deliv. Rev. 106, 27–44. 10.1016/j.addr.2016.09.007 27641944

[ref90] Hu S. Kong J. Sun Z. Han L. Kong W. Yang P. (2011). Heterologous protein display on the cell surface of lactic acid bacteria mediated by the s-layer protein. Microb. Cell Fact. 10:86. 10.1186/1475-2859-10-86, PMID: 22035337 PMC3215925

[ref91] Hu Y. Liu C. Muyldermans S. (2017). Nanobody-based delivery systems for diagnosis and targeted tumor therapy. Front. Immunol. 8:1442. 10.3389/fimmu.2017.01442 29163515 PMC5673844

[ref92] Huet H. A. Growney J. D. Johnson J. A. Li J. Bilic S. Ostrom L.. (2014). Multivalent nanobodies targeting death receptor 5 elicit superior tumor cell killing through efficient caspase induction. MAbs 6, 1560–1570. 10.4161/19420862.2014.975099, PMID: 25484045 PMC4623017

[ref93] Hultberg A. Temperton N. J. Rosseels V. Koenders M. Gonzalez-Pajuelo M. Schepens B.. (2011). Llama-derived single domain antibodies to build multivalent, superpotent and broadened neutralizing anti-viral molecules. PLoS One 6:e17665. 10.1371/journal.pone.0017665, PMID: 21483777 PMC3069976

[ref94] Hussack G. Arbabi-Ghahroudi M. van Faassen H. Songer J. G. Ng K. K. MacKenzie R.. (2011). Neutralization of *Clostridium difficile* toxin A with single-domain antibodies targeting the cell receptor binding domain. J. Biol. Chem. 286, 8961–8976. 10.1074/jbc.M110.198754, PMID: 21216961 PMC3058971

[ref95] Iezzi M. E. Policastro L. Werbajh S. Podhajcer O. Canziani G. A. (2018). Single-domain antibodies and the promise of modular targeting in cancer imaging and treatment. Front. Immunol. 9:273. 10.3389/fimmu.2018.00273 29520274 PMC5827546

[ref96] Isolauri E. Salminen S. Ouwehand A. C. (2004). Microbial-gut interactions in health and disease. Probiotics. Best Pract. Res. Clin. Gastroenterol. 18, 299–313. 10.1016/j.bpg.2003.10.006, PMID: 15123071

[ref97] Kaakoush N. O. Castaño-Rodríguez N. Mitchell H. M. Man S. M. (2015). Global epidemiology of *Campylobacter* infection. Clin. Microbiol. Rev. 28, 687–720. 10.1128/CMR.00006-15, PMID: 26062576 PMC4462680

[ref98] Kajikawa A. Satoh E. Leer R. J. Yamamoto S. Igimi S. (2007). Intragastric immunization with recombinant *Lactobacillus casei* expressing flagellar antigen confers antibody-independent protective immunity against *Salmonella enterica* serovar Enteritidis. Vaccine 25, 3599–3605. 10.1016/j.vaccine.2007.01.055, PMID: 17287050 PMC7115604

[ref99] Kazemi-Lomedasht F. Behdani M. Bagheri K. P. Habibi-Anbouhi M. Abolhassani M. Arezumand R. (2015). Inhibition of angiogenesis in human endothelial cell using VEGF specific nanobody. Mol. Immunol. 65, 58–67. 10.1016/j.molimm.2015.01.010 25645505

[ref100] Kijanka M. Dorresteijn B. Oliveira S. van Bergen en Henegouwen P. M. P. (2015). Nanobody-based cancer therapy of solid tumors. Nanomedicine 10, 161–174. 10.2217/nnm.14.178, PMID: 25597775

[ref101] Klaenhammer T. Altermann E. Arigoni F. Bolotin A. Breidt F. Broadbent J.. (2002). Discovering lactic acid bacteria by genomics. Antonie Van Leeuwenhoek 82, 29–58. 10.1023/A:1020638309912, PMID: 12369195

[ref102] Kong D.-H. Kim M. R. Jang J. H. Na H.-J. Lee S. (2017). A review of anti-angiogenic targets for monoclonal antibody cancer therapy. Int. J. Mol. Sci. 18:1786. 10.3390/ijms18081786, PMID: 28817103 PMC5578174

[ref103] Koromyslova A. D. Hansman G. S. (2015). Nanobody binding to a conserved epitope promotes norovirus particle disassembly. J. Virol. 89, 2718–2730. 10.1128/JVI.03176-14, PMID: 25520510 PMC4325747

[ref104] Koromyslova A. D. Hansman G. S. (2017). Nanobodies targeting norovirus capsid reveal functional epitopes and potential mechanisms of neutralization. PLoS Pathog. 13:e1006636. 10.1371/journal.ppat.1006636, PMID: 29095961 PMC5667739

[ref105] Kruger C. Hu Y. Pan Q. Marcotte H. Hultberg A. Delwar D.. (2002). *In situ* delivery of passive immunity by lactobacilli producing single-chain antibodies. Nat. Biotechnol. 20, 702–706. 10.1038/nbt0702-702, PMID: 12089555

[ref106] Kruger C. Hultberg A. Marcotte H. Hermans P. Bezemer S. Frenken L. G.. (2006). Therapeutic effect of llama derived VHH fragments against *Streptococcus mutans* on the development of dental caries. Appl. Microbiol. Biotechnol. 72, 732–737. 10.1007/s00253-006-0347-0 , PMID: 16636830

[ref107] Kruger C. Hultberg A. van Dollenweerd C. Marcotte H. Hammarstrom L. (2005). Passive immunization by *Lactobacilli* expressing single-chain antibodies against *Streptococcus mutans*. Mol. Biotechnol. 31, 221–231. 10.1111/j.1834-7819.2005.tb00370.x, PMID: 16230772

[ref108] La-Beck N. M. Jean G. W. Huynh C. Alzghari S. K. Lowe D. B. (2015). Immune checkpoint inhibitors: new insights and current place in cancer therapy. Pharmacotherapy 35**,** 963–976. 10.1002/phar.1643, PMID: 26497482

[ref109] Landete J. M. (2017). A review of food-grade vectors in lactic acid bacteria: from the laboratory to their application. Crit. Rev. Biotechnol. 37**,** 296–308. 10.3109/07388551.2016.1144044 26918754

[ref110] Lauwereys M. Arbabi Ghahroudi M. Desmyter A. Kinne J. Holzer W. De Genst E.. (1998). Potent enzyme inhibitors derived from dromedary heavy-chain antibodies. EMBO J. 17**,** 3512–3520. 10.1093/emboj/17.13.3512, PMID: 9649422 PMC1170688

[ref111] Le D. T. Durham J. N. Smith K. N. Wang H. Bartlett B. R. Aulakh L. K.. (2017). Mismatch-repair deficiency predicts response of solid tumors to PD-1 blockade. Science (New York, N.Y.) 357, 409–413. 10.1126/science.aan6733, PMID: 28596308 PMC5576142

[ref112] Le D. T. Uram J. N. Wang H. Bartlett B. R. Kemberling H. Eyring A. D.. (2015). PD-1 blockade in tumors with mismatch-repair deficiency. N. Engl. J. Med. 372, 2509–2520. 10.1056/NEJMoa1500596, PMID: 26028255 PMC4481136

[ref113] Lee P. Abdul-Wahid A. Faubert G. M. (2009). Comparison of the local immune response against *Giardia lamblia* cyst wall protein 2 induced by recombinant *Lactococcus lactis* and *Streptococcus gordonii*. Microbes Infect. 11, 20–28. 10.1016/j.micinf.2008.10.002, PMID: 18992359

[ref114] Leow C. H. Fischer K. Leow C. Y. Cheng Q. Chuah C. McCarthy J. (2017). Single domain antibodies as new biomarker detectors. Diagnostics 7:52. 10.3390/diagnostics7040052, PMID: 29039819 PMC5745390

[ref115] Li X. Xing Y. Guo L. Lv X. Song H. Xi T. (2014). Oral immunization with recombinant *Lactococcus lactis* delivering a multi-epitope antigen CTB-UE attenuates *Helicobacter pylori* infection in mice. Pathog. Dis. 72, 78–86. 10.1111/2049-632x.12173, PMID: 24687988

[ref116] Lievin-Le Moal V. Servin A. L. (2014). Anti-infective activities of *Lactobacillus* strains in the human intestinal microbiota: from probiotics to gastrointestinal anti-infectious biotherapeutic agents. Clin. Microbiol. Rev. 27, 167–199. 10.1128/cmr.00080-13 , PMID: 24696432 PMC3993101

[ref117] Lin C. F. Lo T. C. Kuo Y. C. Lin T. H. (2013). Stable integration and expression of heterologous genes in several lactobacilli using an integration vector constructed from the integrase and *attP* sequences of phage PhiAT3 isolated from *Lactobacillus casei* ATCC 393. Appl. Microbiol. Biotechnol. 97, 3499–3507. 10.1007/s00253-012-4393-5, PMID: 23064454

[ref118] Lin T. H. Lin C. H. Pan T. M. (2018). The implication of probiotics in the prevention of dental caries. Appl. Microbiol. Biotechnol. 102, 577–586. 10.1007/s00253-017-8664-z, PMID: 29192351

[ref119] Linares D. M. Alvarez-Sieiro P. del Rio B. Ladero V. Redruello B. Martin M. C. (2015). Implementation of the agmatine-controlled expression system for inducible gene expression in *Lactococcus lactis*. Microb. Cell Fact. 14:208. 10.1186/S12934-015-0399-X 26715338 PMC4696319

[ref120] Linares D. M. Perez M. Ladero V. Del Rio B. Redruello B. Martin M. C. (2014). An agmatine-inducible system for the expression of recombinant proteins in *Enterococcus faecalis*. Microb. Cell Fact. 13:169. 10.1186/s12934-014-0169-1 25471381 PMC4265343

[ref121] Lipson E. J. (2013). Re-orienting the immune system: durable tumor regression and successful re-induction therapy using anti-PD1 antibodies. Oncoimmunology 2:e23661. 10.4161/onci.23661 23734322 PMC3654592

[ref122] Liu H. Wang Y. Duan H. Zhang A. Liang C. Gao J.. (2015). An intracellularly expressed Nsp9-specific nanobody in MARC-145 cells inhibits porcine reproductive and respiratory syndrome virus replication. Vet. Microbiol. 181, 252–260. 10.1016/j.vetmic.2015.10.021, PMID: 26525739

[ref123] Liu J. K. Hou X. L. Wei C. H. Yu L. Y. He X. J. Wang G. H.. (2009). Induction of immune responses in mice after oral immunization with recombinant *Lactobacillus casei* strains expressing enterotoxigenic *Escherichia coli* F41 fimbrial protein. Appl. Environ. Microbiol. 75**,** 4491–4497. 10.1128/aem.02672-08, PMID: 19447955 PMC2704837

[ref124] Liu S. Li Y. Deng B. Xu Z. (2016). Recombinant *Lactococcus lactis* expressing porcine insulin-like growth factor I ameliorates DSS-induced colitis in mice. BMC Biotechnol. 16:25. 10.1186/s12896-016-0255-z 26932768 PMC4774141

[ref125] Liu Y. Huang H. (2018). Expression of single-domain antibody in different systems. Appl. Microbiol. Biotechnol. 102, 539–551. 10.1007/s00253-017-8644-3 , PMID: 29177623

[ref126] Ma L. Gu K. Zhang C. H. Chen X. T. Jiang Y. Melcher K.. (2016). Generation and characterization of a human nanobody against VEGFR-2. Acta Pharmacol. Sin. 37, 857–864. 10.1038/aps.2016.2, PMID: 27108602 PMC4954766

[ref127] Maffey L. Vega C. G. Parreno V. Garaicoechea L. (2015). Controlling Rotavirus-associated diarrhea: could single-domain antibody fragments make the difference? Rev. Argent. Microbiol. 47, 368–379. 10.1016/j.ram.2015.09.005, PMID: 26654700

[ref128] Malanovic N. Lohner K. (2016). Gram-positive bacterial cell envelopes: the impact on the activity of antimicrobial peptides. Biochim. Biophys. Acta 1858, 936–946. 10.1016/j.bbamem.2015.11.004 26577273

[ref129] Mathiesen G. Sveen A. Piard J. C. Axelsson L. Eijsink V. G. (2008). Heterologous protein secretion by *Lactobacillus plantarum* using homologous signal peptides. J. Appl. Microbiol. 105, 215–226. 10.1111/j.1365-2672.2008.03734.x, PMID: 18298538

[ref130] Mao R. Wu D. Wang Y. (2016). Surface display on lactic acid bacteria without genetic modification: strategies and applications. Appl. Microbiol. Biotechnol. 100, 9407–9421. 10.1007/s00253-016-7842-8, PMID: 27649963

[ref131] Mamat U. Wilke K. Bramhill D. Schromm A. B. Lindner B. Kohl T. A. (2015). Detoxifying *Escherichia coli* for endotoxin-free production of recombinant proteins. Microb. Cell Fact. 14:57. 10.1186/s12934-015-0241-5 25890161 PMC4404585

[ref132] Marcobal A. Liu X. Zhang W. Dimitrov A. S. Jia L. Lee P. P.. (2016). Expression of human immunodeficiency virus Type 1 neutralizing antibody fragments using human vaginal *Lactobacillus*. AIDS Res. Hum. Retrovir. 32, 964–971. 10.1089/aid.2015.0378, PMID: 26950606 PMC5067876

[ref133] Marcotte H. Koll-Klais P. Hultberg A. Zhao Y. Gmur R. Mandar R.. (2006). Expression of single-chain antibody against RgpA protease of *Porphyromonas gingivalis* in *Lactobacillus*. J. Appl. Microbiol. 100, 256–263. 10.1111/j.1365-2672.2005.02786.x, PMID: 16430501

[ref134] Marelli B. Perez A. R. Banchio C. de Mendoza D. Magni C. (2011). Oral immunization with live *Lactococcus lactis* expressing rotavirus VP8 subunit induces specific immune response in mice. J. Virol. Methods 175, 28–37. 10.1016/j.jviromet.2011.04.011, PMID: 21530589

[ref135] Marshall B. J. Barrett L. J. Prakash C. McCallum R. W. Guerrant R. L. (1990). Urea protects *Helicobacter* (*Campylobacter*) *pylori* from the bactericidal effect of acid. Gastroenterology 99, 697–702. 10.1016/0016-5085(90)90957-3, PMID: 2379775

[ref136] Martin M. C. Alonso J. C. Suarez J. E. Alvarez M. A. (2000). Generation of food-grade recombinant lactic acid bacterium strains by site-specific recombination. Appl. Environ. Microbiol. 66, 2599–2604. 10.1128/AEM.66.6.2599-2604.2000 , PMID: 10831443 PMC110586

[ref137] Martin M. C. Pant N. Ladero V. Gunaydin G. Andersen K. K. Alvarez B. (2011). Integrative expression system for delivery of antibody fragments by lactobacilli. Appl. Environ. Microbiol. 77, 2174–2179. 10.1128/AEM.02690-10 21257814 PMC3067310

[ref138] Mays Z. J. Nair N. U. (2018). Synthetic biology in probiotic lactic acid bacteria: at the frontier of living therapeutics. Curr. Opin. Biotechnol. 53, 224–231. 10.1016/j.copbio.2018.01.028, PMID: 29550614 PMC6139064

[ref139] McFarland L. V. Ship N. Auclair J. Millette M. (2018). Primary prevention of *Clostridium difficile* infections with a specific probiotic combining *Lactobacillus acidophilus*, *L. casei*, and *L. rhamnosus* strains: assessing the evidence. J. Hosp. Infect. 99, 443–452. 10.1016/j.jhin.2018.04.017, PMID: 29702133

[ref140] Medina E. Guzman C. A. (2001). Use of live bacterial vaccine vectors for antigen delivery: potential and limitations. Vaccine 19, 1573–1580. 10.1016/S0264-410X(00)00354-6, PMID: 11166877

[ref141] Mejias M. P. Hiriart Y. Lauche C. Fernandez-Brando R. J. Pardo R. Bruballa A. (2016). Development of camelid single chain antibodies against Shiga toxin type 2 (Stx2) with therapeutic potential against Hemolytic Uremic Syndrome (HUS). Sci. Rep. 6:24913. 10.1038/srep24913 27118524 PMC4847011

[ref142] Michon C. Kuczkowska K. Langella P. Eijsink V. G. Mathiesen G. Chatel J. M. (2015). Surface display of an anti-DEC-205 single chain Fv fragment in *Lactobacillus plantarum* increases internalization and plasmid transfer to dendritic cells *in vitro* and *in vivo*. Microb. Cell Fact. 14:95. 10.1186/s12934-015-0290-9 , PMID: 26141059 PMC4491208

[ref143] Michon C. Langella P. Eijsink V. G. Mathiesen G. Chatel J. M. (2016). Display of recombinant proteins at the surface of lactic acid bacteria: strategies and applications. Microb. Cell Fact. 15:70. 10.1186/s12934-016-0468-9 27142045 PMC4855500

[ref144] Mohamadzadeh M. Klaenhammer T. R. (2008). Specific *Lactobacillus* species differentially activate Toll-like receptors and downstream signals in dendritic cells. Expert Rev. Vaccines 7, 1155–1164. 10.1586/14760584.7.8.1155 18844590

[ref145] Monedero V. Rodriguez-Diaz J. Viana R. Buesa J. Perez-Martinez G. (2004). Selection of single-chain antibodies against the VP8* subunit of rotavirus VP4 outer capsid protein and their expression in *Lactobacillus casei*. Appl. Environ. Microbiol. 70**,** 6936–6939. 10.1128/aem.70.11.6936-6939.2004, PMID: 15528568 PMC525132

[ref146] Mukherjee J. Tremblay J. M. Leysath C. E. Ofori K. Baldwin K. Feng X.. (2012). A novel strategy for development of recombinant antitoxin therapeutics tested in a mouse botulism model. PLoS One 7:e29941. 10.1371/journal.pone.0029941, PMID: 22238680 PMC3253120

[ref147] Muyldermans S. (2013). Nanobodies: natural single-domain antibodies. Annu. Rev. Biochem. 82, 775–797. 10.1146/annurev-biochem-063011-092449, PMID: 23495938

[ref148] Muyldermans S. Cambillau C. Wyns L. (2001). Recognition of antigens by single-domain antibody fragments: the superfluous luxury of paired domains. Trends Biochem. Sci. 26, 230–235. 10.1016/S0968-0004(01)01790-X, PMID: 11295555

[ref149] Nakano K. Nomura R. Matsumoto M. Ooshima T. (2010). Roles of oral bacteria in cardiovascular diseases—from molecular mechanisms to clinical cases: cell-surface structures of novel serotype k *Streptococcus mutans* strains and their correlation to virulence. J. Pharmacol. Sci. 113, 120–125. 10.1254/jphs.09R24FM , PMID: 20501965

[ref150] Narita J. Okano K. Kitao T. Ishida S. Sewaki T. Sung M. H.. (2006). Display of alpha-amylase on the surface of *Lactobacillus casei* cells by use of the PgsA anchor protein, and production of lactic acid from starch. Appl. Environ. Microbiol. 72, 269–275. 10.1128/aem.72.1.269-275.2006, PMID: 16391053 PMC1352207

[ref151] Norton P. M. Wells J. M. Brown H. W. Macpherson A. M. Le Page R. W. (1997). Protection against tetanus toxin in mice nasally immunized with recombinant *Lactococcus lactis* expressing tetanus toxin fragment C. Vaccine 15, 616–619. 9178460 10.1016/s0264-410x(96)00241-1

[ref152] Oh Y. Varmanen P. Han X. Y. Bennett G. Xu Z. Lu T.. (2007). *Lactobacillus plantarum* for oral peptide delivery. Oral Microbiol. Immunol. 22, 140–144. 10.1111/j.1399-302X.2007.00338.x, PMID: 17311639

[ref153] O’Neil B. H. Wallmark J. M. Lorente D. Elez E. Raimbourg J. Gomez-Roca C.. (2017). Safety and antitumor activity of the anti-PD-1 antibody pembrolizumab in patients with advanced colorectal carcinoma. PLoS One 12:e0189848. 10.1371/journal.pone.0189848, PMID: 29284010 PMC5746232

[ref154] Oggioni M. R. Beninati C. Boccanera M. Medaglini D. Spinosa M. R. Maggi T. (2001). Recombinant *Streptococcus gordonii* for mucosal delivery of a scFv microbicidal antibody. Int. Rev. Immunol. 20, 275–287. 10.3109/08830180109043039 11878770

[ref155] Oliveira S. Heukers R. Sornkom J. Kok R. J. van Bergen en Henegouwen P. M. P. (2013). Targeting tumors with nanobodies for cancer imaging and therapy. J. Control. Release 172, 607–617. 10.1016/j.jconrel.2013.08.298, PMID: 24035975

[ref156] Olivera-Severo D. Uberti A. F. Marques M. S. Pinto M. T. Gomez-Lazaro M. Figueiredo C. (2017). A new role for *Helicobacter pylori* urease: contributions to angiogenesis. Front. Microbiol. 8:1883. 10.3389/fmicb.2017.01883 29021786 PMC5623709

[ref157] Ouwehand A. C. Salminen S. Isolauri E. (2002). Probiotics: an overview of beneficial effects. Antonie Van Leeuwenhoek 82, 279–289. 10.1023/a:1020620607611 12369194

[ref158] Pant N. Hultberg A. Zhao Y. Svensson L. Pan-Hammarstrom Q. Johansen K.. (2006). Lactobacilli expressing variable domain of llama heavy-chain antibody fragments (lactobodies) confer protection against rotavirus-induced diarrhea. J. Infect. Dis. 194, 1580–1588. 10.1086/508747, PMID: 17083044

[ref159] Pant N. Marcotte H. Hermans P. Bezemer S. Frenken L. Johansen K.. (2011). Lactobacilli producing bispecific llama-derived anti-rotavirus proteins in vivo for rotavirus-induced diarrhea. Future Microbiol. 6, 583–593. 10.2217/fmb.11.32 , PMID: 21585264

[ref160] Pardoll D. M. (2012). The blockade of immune checkpoints in cancer immunotherapy. Nat. Rev. Cancer 12, 252–264. 10.1038/nrc3239, PMID: 22437870 PMC4856023

[ref161] Piñero-Lambea C. Ruano-Gallego D. Fernandez L. A. (2015). Engineered bacteria as therapeutic agents. Curr. Opin. Biotechnol. 35, 94–102. 10.1016/j.copbio.2015.05.004, PMID: 26070111

[ref162] Ren C. Zhang Q. Wang G. Ai C. Hu M. Liu X.. (2014). Modulation of peanut-induced allergic immune responses by oral lactic acid bacteria-based vaccines in mice. Appl. Microbiol. Biotechnol. 98, 6353–6364. 10.1007/s00253-014-5678-7, PMID: 24770368

[ref163] Riazi A. Strong P. C. Coleman R. Chen W. Hirama T. van Faassen H.. (2013). Pentavalent single-domain antibodies reduce *Campylobacter jejuni* motility and colonization in chickens. PLoS One 8:e83928. 10.1371/journal.pone.0083928, PMID: 24391847 PMC3877120

[ref164] Ricci A. Allende A. Bolton D. Chemaly M. Davies R. Girones R. (2018). EFSA panel on biological hazards (EFSA BIOHAZ Panel). Update of the list of QPS-recommended biological agents intentionally added to food or feed as notified to EFSA 7: suitability of taxonomic units notified to EFSA until September 2017. EFSA J. 16:e05131. 10.2903/j.efsa.2018.5131 PMC7328878 32625678

[ref165] Riera-Montes M. O’Ryan M. Verstraeten T. (2017). Norovirus and rotavirus disease severity in children: systematic review and meta-analysis. Pediatr. Infect. Dis. J. 37, 501–505. 10.1097/inf.0000000000001824 29135827

[ref166] Robinson K. Chamberlain L. M. Schofield K. M. Wells J. M. Le Page R. W. (1997). Oral vaccination of mice against tetanus with recombinant *Lactococcus lactis*. Nat. Biotechnol. 15, 653–657. 10.1038/nbt0797-653, PMID: 9219268

[ref167] Rozan C. Cornillon A. Petiard C. Chartier M. Behar G. Boix C.. (2013). Single-domain antibody-based and linker-free bispecific antibodies targeting FcgammaRIII induce potent antitumor activity without recruiting regulatory T cells. Mol. Cancer Ther. 12**,** 1481–1491. 10.1158/1535-7163.mct-12-1012, PMID: 23757164

[ref168] Saerens D. Stijlemans B. Baral T. N. Nguyen Thi G. T. Wernery U. Magez S.. (2008). Parallel selection of multiple anti-infectome nanobodies without access to purified antigens. J. Immunol. Methods 329, 138–150. 10.1016/j.jim.2007.10.005, PMID: 17996887

[ref169] Saez-Lara M. J. Gomez-Llorente C. Plaza-Diaz J. Gil A. (2015). The role of probiotic lactic acid bacteria and bifidobacteria in the prevention and treatment of inflammatory bowel disease and other related diseases: a systematic review of randomized human clinical trials. Biomed. Res. Int. 2015:505878. 10.1155/2015/505878, PMID: 25793197 PMC4352483

[ref170] Salminen S. Nybom S. Meriluoto J. Collado M. C. Vesterlund S. El-Nezami H. (2010). Interaction of probiotics and pathogens—benefits to human health? Curr. Opin. Biotechnol. 21, 157–167. 10.1016/j.copbio.2010.03.016, PMID: 20413293

[ref171] Sarker S. A. Jakel M. Sultana S. Alam N. H. Bardhan P. K. Chisti M. J.. (2013). Anti-rotavirus protein reduces stool output in infants with diarrhea: a randomized placebo-controlled trial. Gastroenterology 145, 740–748.e8. 10.1053/j.gastro.2013.06.053, PMID: 23831050

[ref172] Saxelin M. Tynkkynen S. Mattila-Sandholm T. de Vos W. M. (2005). Probiotic and other functional microbes: from markets to mechanisms. Curr. Opin. Biotechnol. 16, 204–211. 10.1016/j.copbio.2005.02.003, PMID: 15831388

[ref173] Schneewind O. Mihaylova-Petkov D. Model P. (1993). Cell wall sorting signals in surface proteins of gram-positive bacteria. EMBO J. 12, 4803–4811. 10.1002/j.1460-2075.1993.tb06169.x, PMID: 8223489 PMC413927

[ref174] Schneewind O. Missiakas D. (2014). Sec-secretion and sortase-mediated anchoring of proteins in Gram-positive bacteria. Biochim. Biophys. Acta 1843, 1687–1697. 10.1016/j.bbamcr.2013.11.009 24269844 PMC4031296

[ref175] Shi T. Ma Y. Yu L. Jiang J. Shen S. Hou Y.. (2018). Cancer immunotherapy: a focus on the regulation of immune checkpoints. Int. J. Mol. Sci. 19, 1389–1403. 10.3390/ijms19051389, PMID: 29735917 PMC5983802

[ref176] Shibasaki S. Karasaki M. Tafuku S. Aoki W. Sewaki T. Ueda M. (2014). Oral immunization against candidiasis using *Lactobacillus casei* displaying enolase 1 from *Candida albicans*. Sci. Pharm. 82, 697–708. 10.3797/scipharm.1404-07, PMID: 25853077 PMC4318230

[ref177] Shiroza T. Shinozaki-Kuwahara N. Hayakawa M. Shibata Y. Hashizume T. Fukushima K. (2003). Production of a single-chain variable fraction capable of inhibiting the *Streptococcus mutans* glucosyltransferase in *Bacillus brevis*: construction of a chimeric shuttle plasmid secreting its gene product. Biochim. Biophys. Acta 1626, 57–64. 10.1016/s0167-4781(03)00038-1 12697330

[ref178] Siegel S. D. Reardon M. E. Ton-That H. (2017). Anchoring of LPXTG-like proteins to the gram-positive cell wall envelope. Curr. Top. Microbiol. Immunol. 404, 159–175. 10.1007/82_2016_8, PMID: 27097813

[ref179] Sindhu K. N. C. Sowmyanarayanan T. V. Paul A. Babji S. Ajjampur S. S. R. Priyadarshini S.. (2014). Immune response and intestinal permeability in children with acute gastroenteritis treated with *Lactobacillus rhamnosus* GG: a randomized, double-blind, placebo-controlled trial. Clin. Infect. Dis. Off. Publ. Infect. Dis. Soc. Am. 58, 1107–1115. 10.1093/cid/ciu065, , PMID: 24501384 PMC3967829

[ref180] Singh P. P. Sharma P. K. Krishnan G. Lockhart A. C. (2015). Immune checkpoints and immunotherapy for colorectal cancer. Gastroenterol. Rep. (Oxf) 3, 289–297. 10.1093/gastro/gov053, PMID: 26510455 PMC4650981

[ref181] Siontorou C. G. (2013). Nanobodies as novel agents for disease diagnosis and therapy. Int. J. Nanomedicine 8**,** 4215–4227. 10.2147/IJN.S39428, PMID: 24204148 PMC3818023

[ref182] Slordahl T. S. Denayer T. Moen S. H. Standal T. Borset M. Ververken C.. (2013). Anti-c-MET nanobody—A new potential drug in multiple myeloma treatment. Eur. J. Haematol. 91, 399–410. 10.1111/ejh.12185, PMID: 23952536

[ref183] Song A. A.-L. In L. L. A. Lim S. H. E. Rahim R. A. (2017). A review on *Lactococcus lactis*: from food to factory. Microb. Cell Fact. 16:55. 10.1186/s12934-017-0669-x 28376880 PMC5379754

[ref184] Stadtmann A. Zarbock A. (2012). CXCR2: from bench to bedside. Front. Immunol. 3:263. 10.3389/fimmu.2012.00263, PMID: 22936934 PMC3426767

[ref185] Steeland S. Vandenbroucke R. E. Libert C. (2016). Nanobodies as therapeutics: big opportunities for small antibodies. Drug Discov. Today 21, 1076–1113. 10.1016/j.drudis.2016.04.003, PMID: 27080147

[ref186] Steidler L. Hans W. Schotte L. Neirynck S. Obermeier F. Falk W.. (2000). Treatment of murine colitis by *Lactococcus lactis* secreting interleukin-10. Science 289**,** 1352–1355. 10.1126/science.289.5483.1352, PMID: 10958782

[ref187] Steidler L. Neirynck S. Huyghebaert N. Snoeck V. Vermeire A. Goddeeris B.. (2003). Biological containment of genetically modified *Lactococcus lactis* for intestinal delivery of human interleukin 10. Nat. Biotechnol. 21, 785–789. 10.1038/nbt840nbt840, PMID: 12808464

[ref188] Su C. Nguyen V. K. Nei M. (2002). Adaptive evolution of variable region genes encoding an unusual type of immunoglobulin in camelids. Mol. Biol. Evol. 19, 205–215. 10.1093/oxfordjournals.molbev.a004073, PMID: 11861879

[ref189] Szatraj K. Szczepankowska A. K. Chmielewska-Jeznach M. (2017). Lactic acid bacteria – promising vaccine vectors: possibilities, limitations, doubts. J. Appl. Microbiol. 123, 325–339. 10.1111/jam.13446, PMID: 28295939 PMC7166332

[ref190] Thueng-in K. Thanongsaksrikul J. Jittavisutthikul S. Seesuay W. Chulanetra M. Sakolvaree Y.. (2014). Interference of HCV replication by cell penetrable human monoclonal scFv specific to NS5B polymerase. MAbs 6, 1327–1339. 10.4161/mabs.29978, PMID: 25517317 PMC4622650

[ref191] Tokuhara D. Alvarez B. Mejima M. Hiroiwa T. Takahashi Y. Kurokawa S.. (2013). Rice-based oral antibody fragment prophylaxis and therapy against rotavirus infection. J. Clin. Invest. 123, 3829–3838. 10.1172/jci70266, PMID: 23925294 PMC3754275

[ref192] Tremblay J. M. Mukherjee J. Leysath C. E. Debatis M. Ofori K. Baldwin K.. (2013). A single VHH-based toxin-neutralizing agent and an effector antibody protect mice against challenge with Shiga toxins 1 and 2. Infect. Immun. 81, 4592–4603. 10.1128/iai.01033-13, PMID: 24082082 PMC3837998

[ref193] Tsai H.-F. Hsu P.-N. (2017). Cancer immunotherapy by targeting immune checkpoints: mechanism of T cell dysfunction in cancer immunity and new therapeutic targets. J. Biomed. Sci. 24:35. 10.1186/s12929-017-0341-0 28545567 PMC5445514

[ref194] Unger M. Eichhoff A. M. Schumacher L. Strysio M. Menzel S. Schwan C. (2015). Selection of nanobodies that block the enzymatic and cytotoxic activities of the binary *Clostridium difficile* toxin CDT. Sci. Rep. 5:7850. 10.1038/srep07850 25597743 PMC4297958

[ref195] van der Linden R. H. Frenken L. G. de Geus B. Harmsen M. M. Ruuls R. C. Stok W. (1999). Comparison of physical chemical properties of llama VHH antibody fragments and mouse monoclonal antibodies. Biochim. Biophys. Acta 1431, 37–46. 10209277 10.1016/s0167-4838(99)00030-8

[ref196] van der Vaart J. M. Pant N. Wolvers D. Bezemer S. Hermans P. W. Bellamy K.. (2006). Reduction in morbidity of rotavirus induced diarrhoea in mice by yeast produced monovalent llama-derived antibody fragments. Vaccine 24, 4130–4137. 10.1016/j.vaccine.2006.02.045, PMID: 16616802

[ref197] van Pijkeren J. P. Barrangou R. (2017). Genome editing of food-grade *Lactobacilli* to develop therapeutic probiotics. Microbiol. Spectr. 5, 1–25. 10.1128/microbiolspec.BAD-0013-2016, PMID: 28959937 PMC5958611

[ref198] Vandenbroucke K. de Haard H. Beirnaert E. Dreier T. Lauwereys M. Huyck L.. (2010). Orally administered *L. lactis* secreting an anti-TNF Nanobody demonstrate efficacy in chronic colitis. Mucosal Immunol. 3, 49–56. 10.1038/mi.2009.116, PMID: 19794409

[ref199] Vandenbroucke K. Hans W. Van Huysse J. Neirynck S. Demetter P. Remaut E.. (2004). Active delivery of trefoil factors by genetically modified *Lactococcus lactis* prevents and heals acute colitis in mice. Gastroenterology 127, 502–513. 10.1053/j.gastro.2004.05.020, PMID: 15300583

[ref200] Vanlandschoot P. Stortelers C. Beirnaert E. Ibañez L. I. Schepens B. Depla E.. (2011). Nanobodies®: new ammunition to battle viruses. Antivir. Res. 92, 389–407. 10.1016/j.antiviral.2011.09.002, PMID: 21939690

[ref201] Vega C. G. Bok M. Vlasova A. N. Chattha K. S. Gomez-Sebastian S. Nunez C.. (2013). Recombinant monovalent llama-derived antibody fragments (VHH) to rotavirus VP6 protect neonatal gnotobiotic piglets against human rotavirus-induced diarrhea. PLoS Pathog. 9:e1003334. 10.1371/journal.ppat.1003334, PMID: 23658521 PMC3642062

[ref202] Venerito M. Vasapolli R. Rokkas T. Malfertheiner P. (2015). *Helicobacter pylori* and gastrointestinal malignancies. Helicobacter 20 (Suppl 1), 36–39. 10.1111/hel.12255 26372823

[ref203] Vesa T. Pochart P. Marteau P. (2000). Pharmacokinetics of *Lactobacillus plantarum* NCIMB 8826, *Lactobacillus fermentum* KLD, and *Lactococcus lactis* MG 1363 in the human gastrointestinal tract. Aliment. Pharmacol. Ther. 14, 823–828. 10.1046/j.1365-2036.2000.00763.x, PMID: 10848668

[ref204] Vosjan M. J. Vercammen J. Kolkman J. A. Stigter-van Walsum M. Revets H. van Dongen G. A. (2012). Nanobodies targeting the hepatocyte growth factor: potential new drugs for molecular cancer therapy. Mol. Cancer Ther. 11, 1017–1025. 10.1158/1535-7163.mct-11-0891, PMID: 22319202

[ref205] Wang J. Mukhtar H. Ma L. Pang Q. Wang X. (2018). VHH antibodies: reagents for mycotoxin detection in food products. Sensors (Basel) 18, 1–13. 10.3390/s18020485, PMID: 29415506 PMC5855929

[ref206] Wang M. Gao Z. Zhang Y. Pan L. (2016). Lactic acid bacteria as mucosal delivery vehicles: a realistic therapeutic option. Appl. Microbiol. Biotechnol. 100, 5691–5701. 10.1007/s00253-016-7557-x, PMID: 27154346

[ref207] Watkins R. R. Bonomo R. A. (2016). Overview: global and local impact of antibiotic resistance. Infect. Dis. Clin. N. Am. 30, 313–322. 10.1016/j.idc.2016.02.001, PMID: 27208761

[ref208] Watterlot L. Rochat T. Sokol H. Cherbuy C. Bouloufa I. Lefevre F.. (2010). Intragastric administration of a superoxide dismutase-producing recombinant *Lactobacillus casei* BL23 strain attenuates DSS colitis in mice. Int. J. Food Microbiol. 144, 35–41. 10.1016/j.ijfoodmicro.2010.03.037, PMID: 20452077

[ref209] Wei C. H. Liu J. K. Hou X. L. Yu L. Y. Lee J. S. Kim C. J. (2010). Immunogenicity and protective efficacy of orally or intranasally administered recombinant *Lactobacillus casei* expressing ETEC K99. Vaccine 28, 4113–4118. 10.1016/j.vaccine.2009.05.088, PMID: 19539580

[ref210] Weiner L. M. Surana R. Wang S. (2010). Monoclonal antibodies: versatile platforms for cancer immunotherapy. Nat. Rev. Immunol. 10, 317–327. 10.1038/nri2744, PMID: 20414205 PMC3508064

[ref211] Wells J. (2011). Mucosal vaccination and therapy with genetically modified lactic acid bacteria. Annu. Rev. Food Sci. Technol. 2**,** 423–445. 10.1146/annurev-food-022510-133640, PMID: 22129390

[ref212] Wells J. M. Mercenier A. (2008). Mucosal delivery of therapeutic and prophylactic molecules using lactic acid bacteria. Nat. Rev. Microbiol. 6, 349–362. 10.1038/nrmicro1840 18345021 PMC7096801

[ref227] Wells J. M. Wilson P. W. Norton P. M. Gasson M. J. Le Page R. W. (1993). Lactococcus lactis: high-level expression of tetanus toxin fragment C and protection against lethal challenge. Mol. Microbiol. 8, 1155–1162. PMID: 8361360 10.1111/j.1365-2958.1993.tb01660.x

[ref213] Wen L. J. Hou X. L. Wang G. H. Yu L. Y. Wei X. M. Liu J. K.. (2012). Immunization with recombinant *Lactobacillus casei* strains producing K99, K88 fimbrial protein protects mice against enterotoxigenic *Escherichia coli*. Vaccine 30, 3339–3349. 10.1016/j.vaccine.2011.08.036, PMID: 21856357

[ref214] Wu Y. Jiang S. Ying T. (2017). Single-domain antibodies as therapeutics against human viral diseases. Front. Immunol. 8:1802. 10.3389/fimmu.2017.01802 29326699 PMC5733491

[ref215] Wyszynska A. Kobierecka P. Bardowski J. Jagusztyn-Krynicka E. K. (2015). Lactic acid bacteria—20 years exploring their potential as live vectors for mucosal vaccination. Appl. Microbiol. Biotechnol. 99, 2967–2977. 10.1007/s00253-015-6498-0, PMID: 25750046 PMC4365182

[ref216] Xu Y. Xiong L. Li Y. Xiong Y. Tu Z. Fu J.. (2015). Citrinin detection using phage-displayed anti-idiotypic single-domain antibody for antigen mimicry. Food Chem. 177, 97–101. 10.1016/j.foodchem.2015.01.007, PMID: 25660863

[ref217] Yang X. Q. Zhao Y. G. Chen X. Q. Jiang B. Sun D. Y. (2013). The protective effect of recombinant *Lactococcus lactis* oral vaccine on a *Clostridium difficile*-infected animal model. BMC Gastroenterol. 13:117. 10.1186/1471-230x-13-117 23865596 PMC3750240

[ref218] Yang Z. Schmidt D. Liu W. Li S. Shi L. Sheng J.. (2014). A novel multivalent, single-domain antibody targeting TcdA and TcdB prevents fulminant *Clostridium difficile* infection in mice. J. Infect. Dis. 210, 964–972. 10.1093/infdis/jiu196, PMID: 24683195 PMC4192054

[ref219] Yao G. Lam K. H. Weisemann J. Peng L. Krez N. Perry K. (2017). A camelid single-domain antibody neutralizes botulinum neurotoxin A by blocking host receptor binding. Sci. Rep. 7:7438. 10.1038/s41598-017-07457-5 28785006 PMC5547058

[ref220] Yuvaraj S. Al-Lahham S. Marreddy R. K. Dijkstra G. Wolken W. A. Lolkema J. S.. (2008). Human scFv SIgA expressed on *Lactococcus lactis* as a vector for the treatment of mucosal disease. Mol. Nutr. Food Res. 52, 913–920. 10.1002/mnfr.200700132, PMID: 18504703

[ref221] Zabana Y. Domenech E. Manosa M. Garcia-Planella E. Bernal I. Cabre E.. (2010). Infliximab safety profile and long-term applicability in inflammatory bowel disease: 9-year experience in clinical practice. Aliment. Pharmacol. Ther. 31, 553–560. 10.1111/j.1365-2036.2009.04206.x, PMID: 20002026

[ref222] Zadravec P. Strukelj B. Berlec A. (2015). Heterologous surface display on lactic acid bacteria: non-GMO alternative? Bioengineered 6, 179–183. 10.1080/21655979.2015.1040956, PMID: 25880164 PMC4601214

[ref223] Zadravec P. Mavric A. Bogovic Matijasic B. Strukelj B. Berlec A. (2014). Engineering BmpA as a carrier for surface display of IgG-binding domain on *Lactococcus lactis*. Protein Eng. Des. Sel. 27, 21–27. 10.1093/protein/gzt059 24336343

[ref224] Zhang F. Wei H. Wang X. Bai Y. Wang P. Wu J.. (2017). Structural basis of a novel PD-L1 nanobody for immune checkpoint blockade. Cell Discov. 3:17004. 10.1038/celldisc.2017.4, PMID: 28280600 PMC5341541

[ref225] Zhang Z. H. Jiang P. H. Li N. J. Shi M. Huang W. (2005). Oral vaccination of mice against rodent malaria with recombinant *Lactococcus lactis* expressing MSP-1(19). World J. Gastroenterol. 11, 6975–6980. 10.3748/wjg.v11.i44.6975, PMID: 16437602 PMC4717040

[ref226] Zou W. Chen L. (2008). Inhibitory B7-family molecules in the tumour microenvironment. Nat. Rev. Immunol. 8, 467–477. 10.1038/nri2326, PMID: 18500231

